# Asymmetry in catalysis by *Thermotoga maritima* membrane-bound pyrophosphatase demonstrated by a nonphosphorus allosteric inhibitor

**DOI:** 10.1126/sciadv.aav7574

**Published:** 2019-05-22

**Authors:** Keni Vidilaseris, Alexandros Kiriazis, Ainoleena Turku, Ayman Khattab, Niklas G. Johansson, Teppo O. Leino, Paula S. Kiuru, Gustav Boije af Gennäs, Seppo Meri, Jari Yli-Kauhaluoma, Henri Xhaard, Adrian Goldman

**Affiliations:** 1 Research Program in Molecular and Integrative Biosciences, University of Helsinki, Helsinki, Finland.; 2 Drug Research Program, Division of Pharmaceutical Chemistry and Technology, Faculty of Pharmacy, University of Helsinki, Helsinki, Finland.; 3 Malaria Research Laboratory, Immunobiology Research Program, Department of Bacteriology and Immunology, Haartman Institute, University of Helsinki, Helsinki, Finland.; 4 School of Biomedical Sciences and Astbury Centre for Structural Molecular Biology, University of Leeds, Leeds, UK.

## Abstract

Membrane-bound pyrophosphatases are homodimeric integral membrane proteins that hydrolyze pyrophosphate into orthophosphates, coupled to the active transport of protons or sodium ions across membranes. They are important in the life cycle of bacteria, archaea, plants, and parasitic protists, but no homologous proteins exist in vertebrates, making them a promising drug target. Here, we report the first nonphosphorus allosteric inhibitor of the thermophilic bacterium *Thermotoga maritima* membrane-bound pyrophosphatase and its bound structure together with the substrate analog imidodiphosphate. The unit cell contains two protein homodimers, each binding a single inhibitor dimer near the exit channel, creating a hydrophobic clamp that inhibits the movement of β-strand 1–2 during pumping, and thus prevents the hydrophobic gate from opening. This asymmetry of inhibitor binding with respect to each homodimer provides the first clear structural demonstration of asymmetry in the catalytic cycle of membrane-bound pyrophosphatases.

## Teaser

Novel inhibitors of membrane-bound pyrophosphatases unlock asymmetric conformations by binding near the exit channel.

## INTRODUCTION

Membrane-bound pyrophosphatases (mPPases) are a family of enzymes that hydrolyze pyrophosphate into two phosphates and couple this reaction with proton and/or sodium transport across the membrane, creating an electrochemical gradient. These enzymes, initially discovered in photosynthetic bacteria and plants (*1*–*3*) and later found in parasitic protists, archaea, and certain species of bacteria, do not occur in animals and humans (*4*, *5*). In various organisms, mPPases are important for survival under diverse stress situations due to energy limitation, such as osmotic stress, anoxia, mineral deficiency, low temperature, and intense light (*6*). In bacteria and archaea, mPPases reside in the cell membrane (*4*), while in protists, algae, and plants, they can also be located in acidocalcisomes, the vacuole, and/or Golgi apparatus (*7*, *8*).

**Fig. 1:**
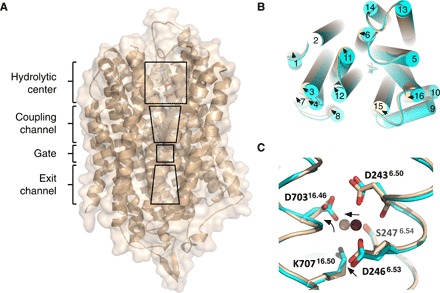
Overview of the TmPPase structure. (**A**) Monomer showing the location of the hydrolytic center, coupling channel, ion gate, and exit channel. (**B**) Top view of the superposition of the TmPPase:IDP:ATC (wheat) and TmPPase:IDP complex (cyan) structure showing relative TMH movements (arrow) upon binding of ATC. (**C**) Superposition of the gate region between two structures [TmPPase:IDP:ATC (wheat) and TmPPase:IDP complex (cyan)]. D246^6.53^, D703^16.46^, and Na^+^ slightly moving away (arrow) relative to their positions in the TmPPase:IDP structure. Violet-purple and pink spheres are for Na^+^ of TmPPase:IDP and TmPPase:IDP:ATC, respectively.

mPPases are homodimeric. Each monomer contains 15 to 17 transmembrane helices (TMHs) with a molecular weight of 70 to 81 kDa. So far, there are structures from just two mPPases: four of *Thermotoga maritima* (TmPPase) and two of mung bean (*Vigna radiata*: VrPPase). TmPPase structures are available in the resting state (TmPPase:Ca:Mg) (*9*), with two phosphates bound (TmPPase:2P_i_) (*9*), with the substrate-analog imidodiphosphate (IDP) bound (TmPPase:IDP) (*10*), and with the phosphate analog (WO_4_) bound (TmPPase:WO_4_) (*10*). VrPPase has been solved in the IDP-bound (VrPPase:IDP) (*11*) and single phosphate-bound states (VrPPase:P_i_) (*10*). In all these structures, mPPase is a symmetric homodimer, with each monomer consisting of 16 TMHs. These helices form two concentric layers, with 6 helices (TMH5, TMH6, TMH11, TMH12, TMH15, and TMH16) forming the inner layer and the other 10 (TMH1 to TMH4, TMH7 to TMH10, TMH13, and TMH14) forming the outer layer. Each monomer consists of four regions: a hydrolytic center, a coupling funnel, an ion gate, and an exit channel (Fig. 1A) (*12*). The hydrolytic center and coupling funnel are composed of TMH5, TMH6, TMH11, TMH12, TMH15, and TMH16 (Fig. 1B). Upon substrate binding, the active site is closed by a long loop between TMH5 and TMH6 and opened again after hydrolysis and ion pumping (*9*–*11*). In the structure of TmPPase:IDP, Na^+^ binds within the membrane plane at the ionic gate between TMH6 and TMH16. Na^+^ is pentacoordinated by the carboxylate groups of D243^6.50^, E246^6.53^, and D703^16.46^, by the O^ϒ^ of S247^6.54^, and with the main-chain carbonyl of D243^6.50^ (Fig. 1C) (*10*). [Residues are numbered as in (*12*), shown as *X*Σ^*a*.*b*^, where *X* is the amino acid, Σ is amino acid position in TmPPase, *a* is the helix number, and *b* is the amino acid position according to a central conserved residue in each helix.]

Parasitic protists such as *Plasmodium falciparum*, *Toxoplasma gondii*, *Trypanosoma brucei*, and *Leishmania donovani* all have H^+^-pumping mPPases (*13*) of two different types (K^+^ dependent and K^+^ independent) in *Plasmodium* (*14*) and of just one type (K^+^ dependent) in the others (*15*–*17*). The mPPases, along with a V–adenosine triphosphatase, maintain the ionic gradient across the acidocalcisome membrane, which is necessary for acidocalcisome function, such as osmotic homeostasis upon passage of the parasite from the insect vector into the mammalian bloodstream (*8*). Knockdown/knockout of mPPases causes severe reduction in polyphosphate and the loss of acidocalcisome acidity, leading to the failure of the parasites to stabilize their intracellular pH upon exposure to external basic pH (*18*). In addition, it has been shown that mPPase is essential for virulence of *T. gondii* in a mouse model (*19*). mPPases are thus promising targets for structure-based drug design because they are essential and do not exist in multicellular animals and structures in different conformations are known.

Nonhydrolyzable PP_i_ analogs are not viable mPPase drug candidates, as they will inhibit the many human enzymes that produce or hydrolyze PP_i_, from inorganic pyrophosphatases to polymerases and ectonucleotide pyrophosphatases/phosphodiesterases (*20*). Our goal is thus the discovery of drug-like small molecular weight compounds that would specifically inhibit mPPases from parasitic protists (*13*). Here, we report the discovery and synthesis of the first nonphosphorus, non–substrate-analog inhibitor of TmPPase, *N*-[(2-amino-6-benzothiazolyl)methyl]-1*H*-indole-2-carboxamide (ATC), its preliminary structure-activity relationships (SARs), and a 3.4 to 4.0 Å resolution structure of ATC bound to TmPPase. It inhibits TmPPase with low micromolar affinity. The binding site of ATC is allosteric and furthermore asymmetric with respect to the structural homodimer, providing strong evidence that the substrate binding and catalysis events proceed asymmetrically.

## RESULTS

### Structure overview

We solved the 3.4 to 4.0 Å resolution of the TmPPase structure in complex with IDP and ATC (TmPPase:IDP:ATC) by molecular replacement using the TmPPase:IDP structure [Protein Data Bank (PDB) ID: 5LZQ] (*10*) as the search model but with the IDP removed. [We quote two numbers for the resolution, as the data are quite anisotropic, as shown by the STARANISO server (http://staraniso.globalphasing.org) with a maximum resolution of 3.6 Å along the a* axis, 3.4 along c*, but slightly worse than 4.0 Å along b* (table S1); we followed current best practice and used all of the data above a local *I*/σ(*I*) cutoff of 3 (*21*).] Molecular replacement identified four TmPPase molecules (two dimers) in the asymmetric unit. After the first round of refinement, we observed positive (*F*_o_-*F*_c_) density at 3.0 σ at the hydrolytic center (fig. S1A) in all four chains and at the ionic gate (fig. S1B) in chains A, B, and D. We built the Mg_5_IDP complex into the former and a sodium ion into the latter. There was positive (*F*_o_-*F*_c_) density between atom O3 of IDP and A495 in chains A, B, and D after placement of the Mg_5_IDP complex. We fit K^+^ into the density (fig. S1A) as this is its known binding site (*11*).

**Fig. 2:**
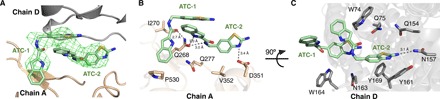
ATC interactions with TmPPase domains. (**A**) ATC [positive (*F*_o_-*F*_c_) electron density map at 3σ] located on the interface of chains A (wheat) and D (gray) in a hydrophobic cleft. Pale green, carbon; blue, nitrogen; red, oxygen; yellow, sulfur. (**B**) Residues in chain A that are important for the interactions with the ATC dimer. The closest approach of the two ATC molecules is 3.3 Å, consistent with π-π stacking. (**C**) Residues on chain D that are important for the interactions with the ATC dimer. Dashed lines represent hydrogen bonds.

We also observed positive 3.0 σ (*F*_o_-*F*_c_) density that was shaped like two connected pancakes near the exit channels of chains A and C. Fitting the density near chain A with ATC in combination with different ions and water molecules (fig. S1, E to J) showed that it was best described as two ATC molecules in a head-to-tail orientation (Fig. 2A and fig. S1E). These ATCs (ATC-**1** and ATC-**2**) are located at the interface between chains A and D (Fig. 2A). However, only one ATC (ATC-**3**) could be located at the interface between chains B and C as the density in these chains was weaker. After refinement, manual rebuilding, and manual placement of the IDP and ATC into the electron density, we were able to refine it to an *R*_work_/*R*_free_ of 22.8%/28.4% with acceptable stereochemistry (table S1). Unlike previously solved TmPPase structures (*9*, *10*), there are four molecules (two dimers, AB and CD) in the asymmetric unit (fig. S2A). The root mean square deviation (RMSD) per Cα is 0.31 Å^2^ for the AB dimer versus the CD dimer and 0.26 to 0.33 Å^2^ when comparing individual monomers (table S2).

There are thus two dimer interfaces in the crystal structure: the AB interface described before (*9*, *10*) and ATC-mediated AD and BC dimers (fig. S2). Are these latter dimers physiologically relevant? It is fairly clear that the answer is no. First, the interactions are weak for a protein interface (249 Å^2^ buried) and the proteins interact tail to tail (fig. S2B), placing the extracellular loops together in a manner that would not allow a bilayer to form. Second, size exclusion chromatography–multiangle laser light scattering (SEC-MALLS) analysis showed that the addition of ATC to the TmPPase:IDP complex (158.9 ± 0.1 kDa) did not change the oligomerization state of the protein (162.8 ± 3.7 kDa for the TmPPase:IDP:ATC complex) (fig. S3). The ATC pair binding near A interacts structurally as a dimer. In the “Kinetics of ATC binding” section, we show that ATC binds functionally as a dimer to both the E_2_S and E_2_S_2_ complexes in solution.

The overall structure of the TmPPase:IDP:ATC complex is very similar to that of the TmPPase:IDP complex, with an RMSD/Cα of 0.50 and 0.43 Å to dimers AB and CD, respectively. (In what follows, all structural alignments were made with respect to monomer A unless otherwise stated.) Although the volume of the hydrolytic pocket in TmPPase:IDP:ATC is very similar to that of TmPPase:IDP (707 Å^3^ versus 720 Å^3^), on the basis of the structural alignment on TMH13 and TMH14 (residues 542 to 629), there are slight clockwise movements on the cytoplasmic side of the protein helices relative to the TmPPase:IDP structure (Fig. 1B). This causes a slight movement of the Mg_5_IDP complex in the same direction as the helices. In addition, at the gate of the TmPPase:IDP:ATC structure, Na^+^ is pentacoordinated by D243^6.50^, E246^6.53^, S247^6.54^, and D703^16.46^ (bidentate to both carboxylate oxygens) (Fig. 1C). In TmPPase:IDP, the coordinating residues are D243^6.50^ (side chain and main chain), E246^6.53^, S247^6.54^, and D703^16.46^, and the D243^6.50^ C═O─Na^+^ distance is 2.8 Å versus the considerably longer 4.0 Å in TmPPase:IDP:ATC. This is mostly because the sodium ion appears to be translated by about ~1.7 Å. We believe that these structural changes are functionally significant and related to the binding of ATC (see the “Asymmetry” section below).

### ATC binds in a cleft beside the exit channel

During refinement, we observed extra density near chains A and D (Fig. 2A), where we were able to model ATC. ATC is a rigid multiring system with only two free torsion angles, which we modeled in trans geometry, the most prevalent in the solution [Supplementary Materials, nuclear magnetic resonance (NMR) spectra]. We could thus (Fig. 2A) place two molecules of ATC into the (*F*_o_-*F*_c_) electron density map. There is (*F*_o_-*F*_c_) density in an equivalent region near chains B and C, where a single ATC could be fitted, but the density there is too poor to fit an ATC dimer, presumably due to molecular disorder in the crystal. We thus focus on ATC-**1** and ATC-**2**, which bind to chains A and D, as representing the TmPPase_2_(Mg_5_PP_i_)_2_(ATC_2_)_1_ complex, where the ATC dimer binds asymmetrically to TmPPase.

**Fig. 3:**
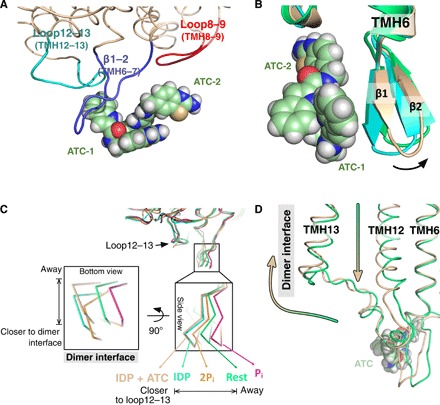
Loop movement caused by the interactions with ATC. (**A**) ATC dimer interaction with β1–2 strand (deep blue), loop8–9 (red), and loop12–13 (cyan). ATC is depicted in space-filling model (pale green, carbon; blue, nitrogen; red, oxygen: yellow, sulfur). (**B**) Superposition of TmPPase:IDP (cyan) with the TmPPase:IDP:ATC structure (wheat) and TmPPase:Ca:Mg (green) showing the large side movement of β1–2 strand (arrow). ATC is depicted as in (A). (**C**) Superposition of TmPPase structures in all different states showing the closer/away movement of β1–2 strand relative to the loop12–13 and the dimer interface of the protein. Coloring scheme from left to right: pink, phosphate analog (WO_4_)–bound state (TmPPase: WO_4_); green, resting state (TmPPase:Ca:Mg); orange, two-product–bound state (TmPPase:2P_i_); cyan, IDP-bound state (TmPPase:IDP); wheat, IDP:ATC-bound state (TmPPase:IDP:ATC). (**D**) Superposition of TmPPase structures showing the downward movement of TMH12 and away movement of loop12–13 from the resting state (green) to the IDP:ATC-bound form (wheat) of TmPPase. The green-to-wheat colored arrow shows the movement.

Two molecules of ATC π-stack head to tail (Fig. 2 and fig. S1D) and bind in a hydrophobic cleft. The cleft is formed by strands β1–2 (loop6–7), loop8–9, and loop12–13, near the exit channel of chain A, with loop2–3 and loop4–5 on chain D (Fig. 3A). (A similar cleft forms between chains B and C.) In terms of buried surface area, ATC-**1** buries 112 Å^2^ on chain A and 85 Å^2^ on chain D, while ATC-**2** buries 85 Å^2^ on chain A and 92 Å^2^ on chain D (table S3).

ATC has a 2-aminothiazole group, which is a weak base [p*K*_a_ (where *K*_a_ is the acid dissociation constant), 4.23 ± 0.03; (*22*)] reported in both neutral and protonated forms in the PDB and CSD (Cambridge Structural Database). We modeled the 2-aminothiazole groups in the protonated state because both ATCs in the AD interface form ion pairs/hydrogen bonds with chain A (Fig. 2B and table S3). Chain A Q268 seems to be the key interacting residue. It forms H bonds to both N11 and N12 of ATC-**2**, and to O01 of ATC-**1** (see fig. S1D for atom numbering of ATC), suggesting how the ATC dimer is stabilized on the enzyme. Chain A D351 (Oδ1) forms a salt bridge with 2-aminothiazole on ATC-**2** (Fig. 2B and table S3). In chain D, on the other hand, the chief interactions do not seem as strong: A single hydrogen bond is formed between ATC-**2** N19 and N157 Oδ1, and the remaining interactions are the hydrophobic stacking of W164 on ATC-**1**, and the indole of ATC-**2** sandwiched between W74 and W164 and stacking on Y161 (Fig. 2C and table S3). These interactions with chain D are probably due to crystal packing, as the binding of ATC to the protein did not change the protein oligomerization in the solution (fig. S3). We therefore focus on the interactions of the ATC dimer with chain A.

### Asymmetry

Artukka and co-workers (*23*) recently demonstrated functional asymmetry in K^+^-dependent mPPases in the presence of excess potassium ion. Here, we show the first structural evidence of asymmetry. The IDP-bound state has the most symmetrical structure (the lowest RMSD values) between each monomer compared to others (table S4 and fig. S4A). However, the binding of ATC makes the IDP-bound enzyme asymmetric, especially in the loops that interact with the inhibitor (loop8–9, β1–2, and loop12–13): The RMSD between the β1–2 loop in chain A (ATC bound) and chain B (no ATC) is 1.25 Å (table S4). Structural alignment between monomers of other states (the resting state, 2P_i_-bound state, and WO_4_^2−^ bound state) shows that these loops differ far less (table S4). In addition, there is a change in the binding mode of Na^+^ at the ionic gate (see above).

ATC binds as a dimer in chain A, holding the β1–2 loop (Q268, K269, I270, and Q277), loop8–9 (D351 and V352), and loop12–13 (P530) together (Fig. 3A and fig. S4, B and C). Comparison of the structure of the loops interacting with ATC with the equivalent loops in TmPPase:IDP showed that the loops β1–2 and 12–13 in chain A move the most, with an RMSD per Cα of 1.13 and 1.09 Å, respectively (table S5), because these loops interact with the ATC dimer (Fig. 3B and table S3). In contrast, the RMSDs/Cα for the other loops that interact with ATC is smaller (table S5). Comparing all the TmPPase states shows that the binding of ATC causes the β1–2 strand of chain A to move away from the dimer interface relative to its position in TmPPase:IDP and TmPPase:2P_i_ to roughly the same angle as in the resting and P_i_ structures (Fig. 3C, inset bottom view; periplasmic side)—when the exit channel must remain closed at all times. The movement of chain A β1–2 due to the binding of the ATC dimer thus creates a hydrophobic clamp that locks the TmPPase exit channel in a closed state after substrate binding. In contrast, it appears that ATC does not bind to chain B because the β1–2 strand has moved away from loop12–13, closer to its position in the resting state (table S4 and fig. S5), and has become disordered. ATC appears not to bind because Q268 and Q277, key elements in the binding site, have become disordered. The same disordering, and lack of binding of ATC, is seen in monomer D.

### Pharmacology of ATC and analogs at TmPPase

**Fig. 4:**
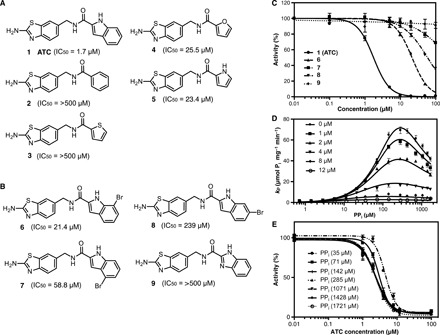
Compound library and inhibition activity against TmPPase. (**A**) Screening hits. (**B**) Analogs developed for structural studies. (**C**) Inhibition curve of ATC, compounds **6** to **9**. (**D**) Kinetic inhibition plot for the inactivation of TmPPase at six different concentrations of ATC. The solid lines represent nonlinear regression fits with a residual SD (Sy.x) of 1.8. (**E**) Concentration-response curve of TmPPase at different ATC and substrate concentrations. The curves are the same for all concentrations of PP_i_ greater than 100 μM (i.e., with significant amounts of the substrate bound) but differ at very low concentrations of the substrate. The enzymatic activity was related to the activity in the absence of the inhibitor (100%), and the background was measured from the signal in the absence of the enzyme. All data are shown as mean ± SD with *n* = 3 replicates.

We synthesized not only ATC but also four analogs (compounds **2**, **3**, **4**, and **5**) (Fig. 4A) with different ring structures. Four other analogs (compounds **6**, **7**, **8**, and **9**), three of them brominated, were also designed for structural studies. The median inhibitory concentrations (IC_50_s) of all the compounds were at least 20 times worse than the original hit ATC (Fig. 4, B and C), and they did not help us solve the structure. They do, however, provide initial SARs for this family of compounds on TmPPase.

The suggested binding mode explains the SARs of the ATC analogs (Fig. 4). First, they show that the hydrogen bonding functionality of the indole ring is important; compounds **2** and **3** that lack this functionality are inactive. In the TmPPase:IDP:ATC structure, Q268 near the indole nitrogen of ATC explains not only the lack of activity of compounds **2** and **3** but also the tolerance of both hydrogen bond donor (nitrogen; compound **5**) and acceptor (oxygen; compound **4**) functionalities in this position. Second, the aromatic nature of the indole ring seems important for activity: Compounds **4** and **5** that include suitable hydrogen bonding functional groups but not a bulky ring structure are approximately 10-fold less active than ATC. The indole rings of ATC**-1** and ATC**-2** form π-π stacking interactions with each other: Removing the benzene ring weakens this interaction. Third, compounds **6**, **7,** and **8** with bromine substitutions are 10- to 100-fold weaker binders than ATC, suggesting the importance of the unsubstituted indole ring. The bromine substitutions may weaken the π-π stacking interactions by altering the shape and location of the π-electron cloud. However, there are also direct clashes with loop6–7 and loop12–13, which are key sites of interaction (see above). In particular, the weakest brominated compound **8** would clash with P530 in loop12–13 (ATC C08-P530: 3.1 to 3.3 Å), while brominated compound **7** would clash with the K269 side chain (ATC C06-K269: 3.8 Å). Last, benzimidazole substitution instead of an indole yields fully inactive compound **9**. This is probably due to the loss of the π-π head-to-tail stacking: Both the 2-aminothiazole and the benzimidazole groups are protonated at physiological pH, so they would repel each other.

### Kinetics of ATC binding

As ATC is a potent inhibitor, we further characterized its effect on the rate of substrate (PP_i_) hydrolysis using a range of ATC concentrations (0.0 to 12.0 μM). We performed the kinetic assay using PP_i_ concentrations from 0 to 1714 μM at 71°C with a single-point measurement at 2 min (Fig. 4D), having shown that this is within the linear range for initial rates (fig. S7). The shape of these curves was unexpected, so we performed a Hill analysis (Fig. 4E and table S6), which showed that, at substrate concentrations greater than 100 μM (i.e., when the substrate is bound; see below), the Hill coefficient for ATC binding is 2.

**Fig. 5:**
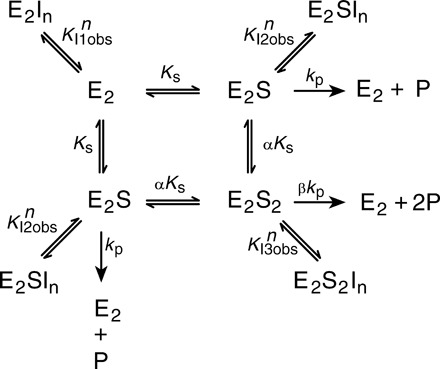
Substrate binding, hydrolysis, and inhibition at two active sites of a dimeric TmPPase.

We therefore performed a simultaneous analysis of the data for all inhibitor and substrate concentrations using the model in Fig. 5 with Eq. 1 and *n = 2* (Table 1), with the exception that *K*_I1_^2^ is not fit (when we included it, the residuals did not improve, and its mean value is ≥0.25 mM; data not shown) (Fig. 4D). The model is a variant on that used by Artukka *et al*. (*23*) and is required as there is half-of-the-sites reactivity. The sum of squares when fitting the inhibition curves with an ATC monomer (i.e., *n* = 1 in Fig. 5) is 5268, and the SD of the residuals (Sy.x) is 4.3; when fitting an ATC dimer, the values are 855 and 1.8, respectively. The model shown (Figs. 4D and 5) is the simplest that fits the data: Other models do not fit (data not shown). We did not endeavor to fit sequential binding of ATC to the TmPPase dimer as the data are insufficient for the purpose. This means that ATC binds as a dimer to TmPPase. ATC binds tightest to the E_2_S complex (*K*_I2obs_ = 1.37 ± 0.09 μM): Its binding to E_2_S_2_ is about 2.5-fold weaker (*K*_I3obs_ = 3.18 ± 0.15 μM), and it does not bind to E_2_ at concentrations we are able to achieve (Table 1). ATC is thus an uncompetitive dimeric inhibitor with a clear preference for binding to the single-substrate bound form.

**Table 1:** Kinetic parameters of TmPPase activity and ATC inhibition constant.

**Kinetic parameter**	**Value**
*k*_p_	235 ± 28 μmol P_i_ mg^–1^ min^–1^
β	0.05 ± 0.005
β*k*_p_	12.2 ± 1.03 μmol P_i_ mg^–1^ min^–1^
*K*_s_	666 ± 110 μM
α	0.12 ± 0.05
α*K*_s_	80.7 ± 19.3 μM
*K*_I2obs_	1.37 ± 0.09 μM
*K*_I3obs_	3.18 ± 0.15 μM

Our data also show that, after one monomer binds the substrate to form the E_2_S complex, the other monomer binds the substrate much more tightly: The value of α is very low (0.12) (Table 1). However, the hydrolysis rate of the E_2_S_2_ complex is 20-fold slower (β = 0.05) compared to the E_2_S complex (*k*_p_ = 234.5 ± 27.1 μmol P_i_ mg^–1^ min^–1^) (Table 1). In comparison with earlier reports (*23*), the binding behavior is different but the ratio of hydrolysis rates is similar (see Discussion). In the crystal structure we solved, ATC is bound to the E_2_S_2_ form, not to the E_2_S form. The concentration of ATC added for crystallization (1 mM) was higher than the binding constant of ATC to the E_2_S_2_ form (Table 1), and ATC binding to the E_2_S form may induce a conformational change in the other monomer that allows further substrate binding (see Discussion). This is consistent with the fact that we could only crystallize the TmPPase:ATC complex in the presence of IDP. ATC is thus the first nonphosphorus inhibitor and the first allosteric inhibitor identified for mPPase.

### Is ATC a viable lead against parasitic mPPases?

ATC would be a potential lead as an antiparasitic agent if it were active against parasitic protists such as *Plasmodium* spp. Preliminary studies with isolated *P. falciparum* membranes showed that ATC had an IC_50_ of around 80 μΜ (fig. S6A), i.e., at least 40-fold lower than in our test system *T. maritima* (Fig. 4A). In the *P. falciparum* survival assays, ATC did not show any anti-plasmodial activity (fig. S6B). On the other hand, knockout of mPPases in *T. gondii* (*19*) and *T. brucei* (*24*) demonstrate that functional mPPase is required for survival and virulence of these parasites.

**Fig. 6:**
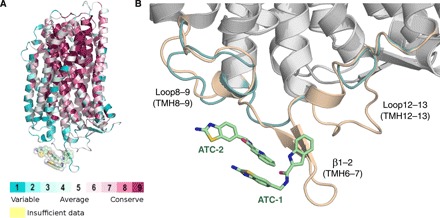
The conservation of ATC binding site among mPPases. (**A**) Structure of TmPPase colored according to the sequence conservation among the set of 16 pathogenic mPPases. The pale green surface indicates the location of the ATC binding site. (**B**) Comparison of the ATC binding sites of the crystal structure of TmPPase (wheat) and a homology model of PfPPase (teal). Carbon atoms of ATC are presented as pale green sticks. Blue, nitrogen; red, oxygen; yellow, sulfur.

To explain these findings and study the potential specificity determinants at the binding sites of ATC-**1** and ATC-**2** across mPPases, we retrieved the sequences of 16 mPPases from parasitic protists as well as representatives of all the other mPPase families (fig. S8). The areas corresponding to loop6–7 (i.e., β1–2 strand), loop8–9, and loop12–13 showed a high degree of difference between TmPPase and the other mPPases (Fig. 6A and fig. S8). In particular, β1–2 (loop6–7) is missing in parasite mPPases. As the binding site of ATC-**1** and ATC-**2** is located on these loops (6–7, 8–9, and 12–13 of chain A), ATC could not bind the mPPases of pathogenic parasites as it does TmPPase. To validate this finding, we built a homology model of *P. falciparum* mPPase (PfPPase; Fig. 6B). Loop6–7 and loop12–13 of the modeled structure are notably shorter than those of TmPPase: The ATC binding site is not present, as loop6–7 contributes about two-thirds of the interface (table S3). This not only explains why ATC does not inhibit PfPPase but also supports the notion that it functions asymmetrically, preventing a full catalytic cycle from occurring by binding to one chain and preventing the exit channel from opening.

## DISCUSSION

### The first novel inhibitors of integral membrane pyrophosphatases

mPPases are potential targets for anti-protozoan drug design as they do not occur in humans and other mammals, are essential in *T. brucei* (*24*), and render *T. gondii* noninfectious (*19*). Bisphosphonate derivatives (PP_i_ analogs) can inhibit mung bean mPPase (*25*) and prevent the proliferation of *T. gondii* (*26*) and other parasitic protists (*27*, *28*). However, they also inhibit human enzymes such as farnesyl pyrophosphate synthase, so they are not viable as anti-parasitic drug candidates [for a review, see (*13*)]. This study reports the first nonphosphorus inhibitors of mPPase, which were identified through a screening process. The best compound (ATC) binds and inhibits both the E_2_S and E_2_S_2_ complexes, with inhibition constants *K*_i2obs_ and *K*_i3obs_ of 1.37 and 3.18 μM, respectively (Table 1). The overall IC_50_ is 1.7 μM. It appears to act in an allosteric manner. This mode of inhibition has been observed for an inhibitor of soluble PPase from *Mycobacterium tuberculosis* (MtsPPase) (*29*), but to our knowledge, this is the first one observed in mPPase.

### Identification of alternate inhibitory mechanisms and evidence for allostery in mPPases

ATC binds beside the exit channel (Figs. 2 and 3) and acts in allosteric manner. Similar types of inhibition occur in GPCRs (G protein–coupled receptors) and many other membrane proteins. For instance, the voltage-dependent K_v_11.1 (hERG) potassium channel that mediates the repolarizing current in the cardiac action potential is easily inhibited by a wide variety of drugs, leading to drug-induced long QT syndrome and lethal arrhythmia. The molecules, such as quinolone and macrolide antibiotics, bind to hydrophobic pockets on the side of the selectivity filter (*30*, *31*). Recently, a structural study found inhibitors that targeted a hitherto-unknown allosteric transmembrane site in MsbA, an adenosine triphosphate (ATP)–binding cassette (ABC) transporter, by locking MsbA in an inward facing conformation that prevents transition to the outward-facing conformation during lipopolysaccharide transport (*32*). This binding uncoupled the nucleotide-binding domains, which adopt an asymmetrical conformation.

Similar interactions to the π-stacked ATC dimer that binds to TmPPase have been observed in soluble protein-inhibitor complexes as well. For example, Stornaiuolo *et al*. (*33*) found an isoquinoline derivative that formed a π-stack of three identical molecules that bound to one binding site of acetylcholine-binding protein. A quinazoline derivative formed a π-stack of five molecules to the lysine-specific demethylase 1 (LSD1)–RE1-silencing transcription factor corepressor (CoRESt) complex (*34*), and a phenylpyrrole derivative forms a head-to-tail π-stacked dimer that binds in the twofold cavity in soluble hexameric MtsPPase. This inhibitor appears to act uncompetitively with the binding site 20 Å from the nearest pyrophosphate (*29*). The asymmetric binding of the ATC dimer to one monomer of TmPPase is similar in kind.

A recent study has shown that mesophilic mPPases show substrate inhibition with increased *K*_m_ and decreased *V* when two molecules of the substrate are bound in the presence of 50 mM K^+^ (*23*). Artukka and co-workers (*23*) observed that S binds more weakly to E_2_S than to E_2_ and that *k*_cat_ for E_2_S_2_ is lower than for E_2_S. They interpreted this to mean that substrate binding to one monomer causes a conformational change in the other that inhibits its interaction with the substrate. For the enzymes they tested, the ratio of *k*_cat_s varies between 2.9 and 16 [table 4 of Artukka *et al.* (*23*)]; our ratio is 20 (Table 1). However, our data differ in that binding of the first substrate molecule appears to be much weaker than that of the second substrate, as α is 0.12 (Table 1), which is the reverse of what they observed. There are two possible explanations: First, they did not study TmPPase, but a range of mesophilic K^+^-dependent mPPases. Second, and we think more likely, our experiments are performed near the temperature optimum of TmPPase (71°C) but in the presence of *n*-dodecyl-β-D-maltopyranoside (DDM), not of the bidentate membrane-spanning lipids found in *T. maritima* (*35*). TmPPase in DDM micelles is almost certainly more conformationally flexible than it is in *T. maritima* lipids, which would weaken binding of the first substrate molecule. If, as both our and the Artukka *et al.* (*23*) data suggest, mPPases are allosteric, then binding of the first substrate molecule would stabilize the structure of the dimer, increasing the affinity for the second substrate molecule and thus explaining the apparent discrepancy.

However, none of this is germane to the interpretation of the ATC inhibition data. Our ATC-bound structure (Figs. 2 and 3) and inhibition data (Fig. 4, D and E; Table 1; and table S6) are similar: An ATC dimer is required to inhibit the TmPPase_2_:(Mg_5_PP_i_)_1–2_ dimer in the presence of the substrate. The second site is unavailable in the substrate-bound form, and neither monomer binds ATC before substrate binds. At very low substrate concentrations (Fig. 4E and table S6), the Hill coefficient is 3, suggesting that a third ATC is required to inhibit the enzyme at concentrations below 100 μM, when the substrate binds poorly in our assay. We suggest that this is also due to the increased flexibility in DDM at 71°C: ATC binding can stabilize substrate binding.

### Correlation between functional and structural data

As the resolution of the structure is only 3.4 to 4.0 Å, how reliable is our interpretation of the structure? The data were sufficient to identify K^+^ in three of the four active sites. This is the first time that they have been observed in a TmPPase structure: The electron density maps are significantly better than 4.0 Å. In addition, the POLDER analysis (fig. S1, K to P) indicates that the ATC fit the density as expected at this resolution.

The link between the structural and functional data then rests on two independent lines of evidence. First, the functional data are consistent only with tight binding to the substrate-bound protein, and we could only obtain crystals with ATC bound in the presence of IDP. Second, the functional data indicate that ATC binds as a dimer, and this is what is observed. Close examination of the AB dimer in our structure further reveals that there are more differences between the two monomers than in any other TmPPase structure (table S4), particularly in loop5–6 and loop12–13. It is possible that the changes we see are due to crystal packing, not due to binding of ATC. However, as solution data show that binding of ATC causes changes that prevent catalysis, we suggest that these changes explain the lack of activity of the ATC-bound enzyme. The alternative hypothesis is that binding of an ATC dimer beside the exit channel in a way that closes it and affects the TmPPase:IDP dimer has nothing to do with observation of uncompetitive inhibition by an ATC dimer in solution. Our suggestion that the crystal and solution data are related is a more parsimonious explanation.

### Mechanism of action of ATC

If our model is correct, then how does ATC inhibit TmPPase? The binding of the dimer to chain A via loop6–7, loop8–9, and loop12–13 creates a hydrophobic clamp that locks the exit channel in the closed state and TMH12 in the down state. The binding appears to be driven by the multidentate interactions of Q268 with both ATC-**1** and ATC-**2**. We posit that the mechanistic implications of these events are as follows. β1 extends from TMH6, which contains D243^6.50^, D246^6.53^, and D247^6.54^, all of which coordinate Na^+^ in the gate. In the ATC-bound structure, the position and coordination of the Na^+^ change. In our current structure, D246^6.53^, D703^16.46^, and Na^+^ move up to 1.7 Å from their positions in the TmPPase:IDP structure (Fig. 1C). This corresponds to a new state that, we believe, keeps the exit channel closed. Our current model (*10*) posits that, during ion pumping, TMH12 moves downward by at least 2 Å (*9*). Comparing the relative positions of the ATC-bound and resting states suggests that this movement of TMH12 makes loop12–13 move toward the dimeric interface of the protein and TMH13 move “up” toward the cytoplasmic side (Fig. 3D). This would induce equal conformational changes in monomer B in TmPPase:IDP. However, ATC binding locks monomer A TMH12 in the “down” state. Because of its “down” position, loop12–13 and TMH13 can still trigger the conformational changes of monomer B into a state that binds the substrate more tightly. However, further catalysis is impossible because monomer B cannot undergo a full catalytic cycle: The substrate can bind, but the full motions required for catalysis are impossible. Diagnostic of these motions is the conformation that leads to binding of ATC. In agreement with the structure, the kinetic data show that ATC dimer only binds to one of the monomers and binds most tightly to the TmPPase_2_(Mg_5_PP_i_) state (Table 1).

The structure also explains substrate inhibition and the requirement for potassium for maximal activity. The amino acid responsible for K^+^ dependency (A495^12.46^ in TmPPase) is located in TMH12, which is directly linked to the dimer interface (loop12–13 and TMH13). We suggest that, in the presence of K^+^, the downward movement of TMH12 upon substrate binding and ion pumping induces conformational changes in the monomer-monomer interface by the “away” movement of loop12–13 and an upward movement of TMH13 (Fig. 3D). This is then propagated to other TMHs. In the absence of K^+^, such coupling does not occur. These changes increase affinity and activity. They also provide a possible structural explanation for the Na^+^/H^+^ pumping enzymes (*23*, *36*), where H^+^ pumping is not inhibited by 100 mM Na^+^ and 50 mM K^+^. One way this might happen is through a conformation similar to that observed here: The binding of PP_i_ to monomer A and pumping of, say, a proton would drive changes in loop12–13 and TMH13 such that the conformation of monomer B became suitable for binding PP_i_ and pumping Na^+^.

### Revised catalytic model

**Fig. 7:**
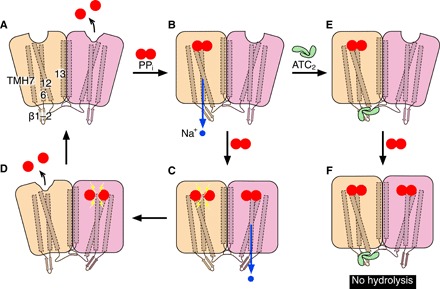
Catalytic scheme of TmPPase and its inhibition by ATC. (**A**) The binding of the substrate (PP_i_; two joined red circles) to monomer A (wheat color) leads to Na^+^ pumping (blue circle and blue arrow for the pumping direction) and induces a conformational change in monomer B (pink color). (**B**) This change increases the affinity of monomer B for the substrate. The Na^+^ pumping causes substrate hydrolysis (yellow lightning symbol for this event) in monomer A, while the substrate binding in monomer B leads to its Na^+^ pumping (**C**). (**D**) After hydrolysis, monomer A releases its hydrolysis products (2P_i_; red circles), while monomer B proceeds to the hydrolysis event, returning the enzyme to state A. The ATC dimer binds to its binding site after substrate binding and Na^+^ pumping in monomer A (**E**), which locks this monomer in the “down” state. Although no hydrolysis can occur in monomer A due to the ATC binding, the conformational change may still induce a conformational change in monomer B, which increases its affinity for the substrate (**F**). The numbering with white background corresponds to TMH number.

In conclusion, we propose the following model for TmPPase catalysis and its inhibition by ATC (Fig. 7). Substrate binding to monomer A leads to pumping in monomer A and induces a conformational change in monomer B, changing its affinity for PP_i_. After PP_i_ binds in monomer B, PP_i_ hydrolysis occurs in monomer A and ion pumping in monomer B. The product of catalysis (2P_i_) is released from monomer A, allowing hydrolysis and subsequent product release in monomer B. The ATC dimer can only bind when PP_i_ is bound to monomer A. Nonetheless, the A:PP_i_ form can still induce the conformational change in monomer B that allows PP_i_ binding. However, since the ATC binding locks monomer A in the down state, no further catalysis can occur.

Our novel structure of TmPPase with IDP and ATC has opened up new routes to drug discovery of neglected diseases by serendipitously demonstrating that there are alternative ways to inhibit mPPase. Although the ATC binding interface is not preserved (Fig. 6 and fig. S8), our data nevertheless indicate that binding at the exit channel can inhibit mPPases. The work shows how extracellular loop binding regions can have significant effects on intracellular substrate binding and vice versa. Last, by stabilizing asymmetric complexes, ATC provides a route into determining other steps along the reaction pathway and thus deriving a full catalytic model—one that may unify the Na^+^/H^+^ and Na^+^ mPPases, as outlined above.

## MATERIALS AND METHODS

### Discovery of ATC and its analogs

The screening process that led to the discovery of compound **1** (ATC) will be described in more detail elsewhere. Briefly, we tested compounds using a recently developed 96-well plate assay with TmPPase as a model system (*37*). The screening collections included compounds synthesized in our laboratories. The identification of ATC as an inhibitor was followed by the test of analogs **2** to **5**. ATC and its analogs were initially developed for their antimicrobial, antiviral, and anticancer activities, as reported elsewhere (*38*).

### Compound synthesis

The purity of all the compounds tested was above 95%. The synthesis of ATC (compound **1**) and compounds **2** to **5** has been described elsewhere (*38*). The synthesis of compounds **6** to **9** is described in the Supplementary Materials. For synthesis, all reactions were carried out using commercially available starting materials (Sigma-Aldrich, Schnelldorf, Germany; BioFine International Inc., Vancouver, Canada; Fluorochem, Hadfield, UK) and solvents without further purification. Column chromatography was performed with an automated Isolera One high-performance flash chromatography system (Biotage, Uppsala, Sweden) using a 0.1-mm path length flow cell ultraviolet (UV) detector/recorder module (fixed wavelength, 254 nm). Analytical thin-layer chromatography was carried out using 0.2-mm silica gel plates (silica gel 60, F_254_; Merck KGaA, Darmstadt, Germany). NMR spectra (^1^H NMR and ^13^C NMR) were recorded on a Bruker Ascend 400 spectrometer (Bruker Corporation, Billerica, MA, USA). ^1^H NMR was measured at 400 MHz, and ^13^C NMR was measured at 100 MHz. High-resolution mass spectra (HRMS) were measured on a Waters Synapt G2 mass spectrometer (Waters Corporation, Milford, MA, USA) and reported for the molecular ions [M + H]^+^.

### Protein expression and purification

Expression and purification of TmPPase are described elsewhere (*39*, *40*). In brief, His-tagged TmPPase in pRS1024 plasmid under the control of the PMA1 promoter was freshly transformed into *Saccharomyces cerevisiae* strain BJ1991. The cells were grown overnight in 250 ml of selective synthetic complete dropout medium and then added to 740 ml of 1.5× YP medium containing 2% glucose. The cells were further grown at 30°C for 8 hours, collected by centrifugation (4000 rpm, 10 min), and lysed at 4°C using a bead beater with 0.2-mm glass beads. The membrane fraction was collected by ultracentrifugation (100,000*g*, 45 min), and the pellets were resuspended in buffer containing 50 mM MES-NaOH (pH 6.5), 20% (v/v) glycerol, 50 mM KCl, 5.2 mM MgCl_2_, 1.33 mM dithiothreitol (DTT), pepstatin A [2 μg ml^−1^ (w/v)] (Sigma), and 0.334 mM phenylmethylsulfonyl fluoride (PMSF; Sigma). The membranes were solubilized using the “hot-solve” method (*40*) at 75°C for 1.5 hours in solubilization buffer [50 mM MES-NaOH (pH 6.5), 20% (v/v) glycerol, and 5.33% (w/v) DDM (Anatrace)]. After centrifugation to remove denatured proteins, KCl (to a final concentration of 0.3 M) and 2 ml of nickel–nitrilotriacetic acid (Ni-NTA) beads (Qiagen) were added and incubated at 40°C for 1.5 hours to each 40 ml of the solubilized protein and then loaded into an Econo-Pac column (Bio-Rad). Then, the column was washed with 2 × CV (column volume) of washing buffer [50 mM MES-NaOH (pH 6.5), 20% (v/v) glycerol, 50 mM KCl, 20 mM imidazole (pH 6.5), 5 mM MgCl_2_, 1 mM DTT, pepstatin A (2 mg ml^−1^, w/v), 0.2 mM PMSF, and 0.05% DDM (Anatrace)] and eluted with 2 × CV of elution buffer [50 mM MES-NaOH (pH 6.5), 3.5% (v/v) glycerol, 50 mM KCl, 400 mM imidazole (pH 6.5), 5 mM MgCl_2_, 1 mM DTT, pepstatin A (2 mg ml^−1^, w/v), 0.2 mM PMSF and 0.5% octyl glucose neopentyl glycol (OGNPG; Anatrace), 1 mM DTT, pepstatin A (2 mg ml^−1^, w/v), and 0.2 mM PMSF and 0.05% DDM (Anatrace)].

### Activity measurement and kinetic analysis

TmPPase activity, compound screening, and kinetic experiments were done using the molybdenum blue reaction method in a purified protein solubilized in DDM (*37*). Initial reaction rates of TmPPase were determined using PP_i_ as the substrate and ATC as the inhibitor at varying concentrations. The concentration of MgCl_2_ and Na_4_PP_i_ required to maintain 5 mM free Mg^2+^ at pH 8.0 was approximated as described previously (*41*). The reaction was done in reaction buffer [60 mM tris-HCl (pH 8.0), 5 mM free Mg^2+^, 100 mM KCl, and 10 mM NaCl] at 71°C for 2 min. We chose a 2-min reaction time as it still produces a reaction product in a linear range (fig. S7).

For analysis, we used a variant of Artukka and co-workers’ method (*23*), which assumes that the two active sites in an mPPase dimer interact allosterically, with the addition of inhibitor binding to the two active sites (Fig. 5). E_2_ represents the dimeric enzyme, *S* is the substrate (Na_4_PP_i_), *I* is the inhibitor (ATC), and *n* is the number of ATC molecules bound to the enzyme. *K*_S_ is a microscopic Michaelis-Menten constant, and *k*_p_ is the per-site maximum rate for the substrate complex. α is the factor relating the binding of the first substrate molecule (*K*_S_) to the binding of the second, β is the factor relating the rate of hydrolysis of the E_2_S complex (*k*_p_) to that of the E_2_S_2_ complex, and *K*_I1obs_, *K*_I2obs_, and *K*_I3obs_ are the observed inhibition constants for binding to the E_2_, E_2_S, and E_2_S_2_ complexes.

For the mechanism in Fig. 5 with *n* = 2 (i.e., only inhibitor dimers bind), the rate Eq. 1 (*42*) is

$$v=\frac{\left(2k_{p}+2\beta k_{p}[S]/\alpha K_{s}\right)}{2(1+{\left[I\right]}^{2}/{\left(K_{I2\text{obs}}\right)}^{2})+(K_{s}/[S])(1+{\left[I\right]}^{2}/{\left(K_{I1\text{obs}}\right)}^{2})+([S]/\alpha K_{s})(1+{\left[I\right]}^{2}/{\left(K_{I3\text{obs}}\right)}^{2})}$$ (1)

where [*S*] and [*I*] correspond to substrate and inhibitor concentrations, respectively, and the equilibrium and Michaelis-type constants are as in Fig. 5. The kinetic data were fit together to obtain all parameter values and their SEs by nonlinear regression as a function of inhibitor (0, 1.0, 2.0, 4.0, 8.0, and 12.0 μM) and substrate (0 to 1714 μM) concentrations using Prism 6.0 (GraphPad Software). Eliminating the *K*_I1_ terms had no effect on the overall quality of fit.

### Crystallization, structure determination, and analysis

For crystallization, the purified protein was buffer-exchanged to the crystallization buffer [50 mM MES-NaOH (pH 6.5), 3.5% (v/v) glycerol, 50 mM KCl, 5 mM MgCl_2_, 2 mM DTT, and 0.5% OGNPG] on a Micro Bio-Spin 6 column (Bio-Rad) and then diluted to a concentration of 10 mg ml^−1^. Before crystallization, 1 mM IDP and 1 mM ATC were added, and the solution was incubated on ice for 10 min and centrifuged for 20 min (16,000*g*, 4°C). Crystallization trials were done at 22°C by sitting-drop vapor diffusion method using commercial screens, MemGold and PGA screen (Molecular Dimensions), in MRC two-well crystallization plates (Swissci) with a mosquito robot (TTP Labtech), and the drops were monitored using a minstrel DT UV imaging system (Formulatrix). Crystal hits appeared in the MemGold screen under different conditions. Crystallization conditions were then optimized to get better crystals by varying pH, buffer, salt, and polyethylene glycol (PEG) concentrations using the vapor diffusion method in 1 μl + 1 μl (protein–mother liquor) drops in a 24-well plate at room temperature. Harvestable crystals appeared within a week and were frozen after soaking in cryoprotectant (mother liquor + 20% glycerol) or directly from mother liquor. The best diffracting crystal appeared from a solution containing 0.1 M MES (pH 6.5), 0.1 M NaCl, 33% PEG-400, and 4% ethylene glycol.

X-ray diffraction data were collected at the European Synchrotron Radiation Facility, Grenoble (France), on the ID29 beamline at 100 K on a PILATUS 6M detector. One thousand four hundred images were collected at an oscillation angle of 0.1°. The data were merged and scaled using X-ray Detector Software (XDS) (*43*). Because of the anisotropy of the diffraction data, anisotropic cutoff and correction of the merged intensity data were performed on the STARANISO server (http://staraniso.globalphasing.org/) using the DEBYE and STARANISO programs. The structure was solved by molecular replacement with Phaser (*44*) using the IDP-bound state of the TmPPase structure (5LZQ) (*10*) as the search model. Phaser found a unique solution in space group *P*2_1_2_1_2_1_, with four monomers in the asymmetric unit forming two dimers. The structure was built and refined using phenix.refine (*45*) and Coot (*46*). The final model had *R*_work_/*R*_free_ of 22.8%/28.4%, with 95.7% of residues in the most favored and 4.3% in the allowed region of the Ramachandran plot (table S1).

The structural interface and assembly of the protein were analyzed using the PDBe PISA server (www.ebi.ac.uk/pdbe/pisa/) (*47*). Internal pocket analysis was done using the SplitPocket server (http://pocket.med.wayne.edu/patch/) (*48*, *49*). Structural alignments were done in PyMOL with the default setting using the monomer structure, except if stated differently. All structural figures were produced using PyMOL (https://pymol.org/2/).

### Comparison with pathogenic mPPases

The sequences of TmPPase and 16 pathogenic mPPases were aligned using Clustal Omega software (*50*), and conservation analysis was conducted and visualized with the ConSurf server (*51*, *52*) with default parameters. The initial pairwise sequence alignment of TmPPase and *P. falciparum* mPPase (VP1; PfPPase; sequence identity, 42%) was produced with Discovery Studio 4.5 software (*53*) using the Align123 algorithm. The sequence alignment figure was generated with ESPript 3.0 (http://espript.ibcp.fr/ESPript/ESPript/index.php) (*54*). Homology modeling was carried out with Modeller 9v14 (*55*) with default settings in the Discovery Studio platform. The pairwise alignment was revised during the course of model building; in total, nine models were constructed in three modeling rounds. The Modeller DOPE scores did not differ significantly among these models, and a representative model was selected to minimize strain in the loops connecting TMHs.

## Supplementary Material

http://advances.sciencemag.org/cgi/content/full/5/5/eaav7574/DC1

Download PDF

## SUPPLEMENTARY MATERIALS

Supplementary material for this article is available at http://advances.sciencemag.org/cgi/content/full/5/5/eaav7574/DC1 Fig. S1. Electron density maps of ligands in the TmPPase:IDP:ATC structure. Fig. S2. Asymmetric unit of TmPPase:IDP:ATC. Fig. S3. Oligomerization state of TmPPase upon the addition of inhibitors. Fig. S4. Comparison of the ATC binding sites in different TmPPase structures. Fig. S5. Differences in the ATC binding site between monomers of the TmPPase:IDP:ATC structure. Fig. S6. Effect of ATC on growth of *P. falciparum* and on PfPPase. Fig. S7. Linearity of TmPPase activity as a function of time and concentration. Fig. S8. Multiple sequence alignment of mPPases from different organisms. Scheme S1. Synthesis of analogs of **1** with heavy and basic atoms. Table S1. X-ray data collection and refinement statistics. Table S2. RMSD of TmPPase monomer in the asymmetric unit relative to monomer A. Table S3. Major interactions of ATC with chains A and D. Table S4. RMSD between chains A and B of loops in different TmPPase structures. Table S5. RMSD of chain A of TmPPase:IDP:ATC loops to chain A of TmPPase:IDP loops. Table S6. Hill constant of TmPPase inhibition by ATC at different substrate concentrations. References (*56*–*58*)
