# Supplementary material for: Asymmetry in catalysis by Thermotoga maritima membrane-bound pyrophosphatase demonstrated by a nonphosphorus allosteric inhibitor
Source: Sci Adv. 2019 May 22;5(5):eaav7574. doi: 10.1126/sciadv.aav7574 (PMC6530997; doi:10.1126/sciadv.aav7574)
Supplement: http://advances.sciencemag.org/cgi/content/full/5/5/eaav7574/DC1 [file supp_5_5_eaav7574__index.html]

Science Advances | Science Advances

## Supplementary Materials

**This PDF file includes:**

- Fig. S1. Electron density maps of ligands in the TmPPase:IDP:ATC structure.
- Fig. S2. Asymmetric unit of TmPPase:IDP:ATC.
- Fig. S3. Oligomerization state of TmPPase upon the addition of inhibitors.
- Fig. S4. Comparison of the ATC binding sites in different TmPPase structures.
- Fig. S5. Differences in the ATC binding site between monomers of the TmPPase:IDP:ATC structure.
- Fig. S6. Effect of ATC on growth of *P. falciparum* and on PfPPase.
- Fig. S7. Linearity of TmPPase activity as a function of time and concentration.
- Fig. S8. Multiple sequence alignment of mPPases from different organisms.
- Scheme S1. Synthesis of analogs of 1 with heavy and basic atoms.
- Table S1. X-ray data collection and refinement statistics.
- Table S2. RMSD of TmPPase monomer in the asymmetric unit relative to monomer A.
- Table S3. Major interactions of ATC with chains A and D.
- Table S4. RMSD between chains A and B of loops in different TmPPase structures.
- Table S5. RMSD of chain A of TmPPase:IDP:ATC loops to chain A of TmPPase:IDP loops.
- Table S6. Hill constant of TmPPase inhibition by ATC at different substrate concentrations.
- References (*56*–*58*)

Download PDF

**Files in this Data Supplement:**

- Adobe PDF - aav7574\_SM.pdf
