# Supplementary material for: Asymmetry in catalysis by Thermotoga maritima membrane-bound pyrophosphatase demonstrated by a nonphosphorus allosteric inhibitor
Source: Sci Adv. 2019 May 22;5(5):eaav7574. doi: 10.1126/sciadv.aav7574 (PMC6530997; doi:10.1126/sciadv.aav7574)
Supplement: Download PDF [file aav7574_SM.pdf]

Science Advances
AAAS

advances.sciencemag.org/cgi/content/full/5/5/eaav7574/DC1

Supplementary Materials for

Asymmetry in catalysis by *Thermotoga maritima* membrane-bound pyrophosphatase demonstrated by a nonphosphorus allosteric inhibitor

Keni Vidilaseris, Alexandros Kiriazis, Ainoleena Turku, Ayman Khattab, Niklas G. Johansson, Teppo O. Leino, Paula S. Kiuru, Gustav Boije af Gennäs, Seppo Meri, Jari Yli-Kauhaluoma, Henri Xhaard, Adrian Goldman*

*Corresponding author. Email: a.goldman@leeds.ac.uk

Published 22 May 2019, *Sci. Adv.* **5**, eaav7574 (2019)
DOI: 10.1126/sciadv.aav7574

This PDF file includes:

Fig. S1. Electron density maps of ligands in the TmPPase:IDP:ATC structure.
Fig. S2. Asymmetric unit of TmPPase:IDP:ATC.
Fig. S3. Oligomerization state of TmPPase upon the addition of inhibitors.
Fig. S4. Comparison of the ATC binding sites in different TmPPase structures.
Fig. S5. Differences in the ATC binding site between monomers of the TmPPase:IDP:ATC structure.
Fig. S6. Effect of ATC on growth of *P. falciparum* and on PfPPase.
Fig. S7. Linearity of TmPPase activity as a function of time and concentration.
Fig. S8. Multiple sequence alignment of mPPases from different organisms.
Scheme S1. Synthesis of analogs of **1** with heavy and basic atoms.
Table S1. X-ray data collection and refinement statistics.
Table S2. RMSD of TmPPase monomer in the asymmetric unit relative to monomer A.
Table S3. Major interactions of ATC with chains A and D.
Table S4. RMSD between chains A and B of loops in different TmPPase structures.
Table S5. RMSD of chain A of TmPPase:IDP:ATC loops to chain A of TmPPase:IDP loops.
Table S6. Hill constant of TmPPase inhibition by ATC at different substrate concentrations.
References (*56–58*)

Supplementary Materials

Supplementary Methods

Size-exclusion chromatography – Multi angle laser light scattering (SEC-MALLS)

50 μg of TmPPase purified in DDM with IDP (1 mM) and IDP+ATC (1 mM each) were run through a Superdex 200 10/300 GL equilibrated with a buffer containing 50 mM MES-NaOH pH 6.5, 50 mM KCl, 5 mM $MgCl_2$, 2 mM DTT and 0.05% DDM using a HPLC-system (Shimadzu). The eluted samples from the column were analysed using UV-absorbance, refractive index (Optilab rEX, Wyatt) and multi angle laser light scattering (miniDAWN TREOS, Wyatt) detectors and the enzyme molecular mass was calculated using ASTRA software (Wyatt).

Anti-plasmodial activity assay

For the *in vitro* anti-plasmodial activity assay, *Plasmodium falciparum* strain 3D7 was maintained in culture as described previously (*56*). Briefly, parasites were cultured in O+ human erythrocytes at 5% haematocrit in RPMI-1640 medium supplemented with 0.5% Albumax II (Gibco, Carlsbad, CA, USA), 200 μM hypoxanthine (Sigma, St. Louis, MO, USA) and 20 μg ml^{-1} gentamycin (Gibco). Parasites were synchronized with the sorbitol method as described (*57*). The test compound was dissolved in DMSO to make a stock solution of 10 mM. Two-fold serial dilutions of the test compound were made in the culture medium to cover the range of 100 μM-100 nM. Fifty microliters of each dilution were mixed in a 96-well plate with 150 μl of 2% haematocrit of 0.5% parasitemia (ring stage). After 72 h incubation at 37 °C, the parasite growth was quantified using fluorescent SYBR Green I®-based assay as described (*58*). The half-maximal inhibitory concentration (IC_{50}) of the test compound was assessed by non-linear regression in Prism 7 (GraphPad Software). The potent anti-malarial drug, artemisinin, was used in parallel as a positive control.

Experimental procedures and characterization data of compounds

All reactions were carried out with commercially available starting materials (Sigma-Aldrich, Schnelldorf, Germany; Biofine Internatonal, Vancouver, Canada) and solvents without further purification. Anhydrous DMF was obtained commercially and all solvents for work-up and chromatography were obtained as HPLC quality and used as received. Column chromatography was performed with Merck 230−400 mesh silica gel or with an automated Biotage high performance flash chromatography Isolera One (Uppsala, Sweden) using a 0.1 mm path length flow cell UV-detector/recorder module (fixed wavelength: 254 nm). Analytical thin layer chromatography (TLC) was carried out using 0.2-mm silica gel plates (silica gel 60, F_{254}, Merck KGaA, Darmstadt, Germany. Nuclear magnetic resonance spectra (^{1}H NMR at 400 MHz and ^{13}C NMR at 100 MHz) were recorded on a Bruker Ascend 400 (Bruker Corporation, Billerica, Massachusetts, USA. The chemical shifts are reported in parts per million (ppm) on the δ scale using tetramethyl silane (TMS) as an internal standard. The coupling constants J are quoted in Hertz (Hz). Data for ^{1}H NMR spectra are reported as follows: chemical shift (multiplicity, integration, coupling constant(s)). The multiplicity was abbreviated as follows: br, broad signal; s, singlet; d, doublet; t, triplet; q, quartet; quin, quintet; dd, doublet of doublets; dt, doublet of triplets; dq, doublet of quartets; m, multiplet; m_c, centered multiplet. High resolution mass spectra (HRMS) were measured on a Waters Synapt G2 (Waters Corporation, Milford, Massachusetts, USA) and reported for the molecular ions $[M+H]^{+}$. LC-MS purity analyses were performed on a Waters Acquity® UPLC system (Waters, Milford MA, USA) attached to an Acquity PDA detector and Waters Synapt G2 HDMS mass spectrometer with an ESI ion source.

Compounds **1–5** were synthesized according to known literature procedures (*38*).

N-[(2-Aminobenzo[*d*]thiazol-6-yl)methyl]-7-bromo-1*H*-indole-2-carboxamide (6)

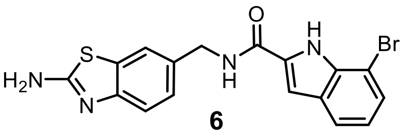

6

6-(Aminomethyl)benzo[*d*]thiazol-2-amine (38 mg, 0.21 mmol), 7-bromoindole-2-carboxylic acid (0.050 g, 0.21 mmol), EDC·HCl (41 mg, 0.21 mmol) and 4-DMAP (25 mg, 0.21 mmol) were dissolved in dry DMF (6 ml) under argon. The resulting light-yellow suspension was stirred at r.t. for 23 h after which TLC from the reaction mixture showed formation of product. The solution was concentrated with the rotary evaporator and the residue was purified by automated column chromatography on silica gel (eluent: EtOAc/*n*-hexane 1:4→3:4) to give **6** as an off-white solid (42 mg, 50%). ^{1}H NMR (400 MHz, DMSO-d_6): δ 11.37 (1H, br s), 9.06 (1H, br t, J = 6.0 Hz), 7.66 (1H, d, J = 6.0 Hz), 7.64 (1H, d, J = 1.6 Hz), 7.44 (1H, dd, J = 0,8 Hz, 7.6 Hz), 7.43 (2H, br s), 7.31 1H, d, J = 8.4 Hz), 7.26 (1H, d, J = 2.0 Hz), 7.21 (1H, dd, J = 1.6 Hz, 8.4 Hz), 7.01 (1H, t, J = 7.6 Hz), 4.53 (2H, d, J = 6.0 Hz); ^{13}C NMR (100 MHz, DMSO-d_6): δ 166.4, 160.0, 152.0, 135.1, 133.2, 131.8, 131.1, 128.4, 126.3, 125.3, 121.3, 121.1, 120.0, 117.5, 106.0, 104.5, 42.4; LC-MS: $[M+H]^+$, *m/z* 401 (t_r = 2.76 min), ≥95%; HRMS-ESI *m/z*: calc. for $C_{17}H_{13}BrN_4OS$ $[M+H]^+$: 401.0072, found 401.0075.

N-[(2-Aminobenzo[*d*]thiazol-6-yl)methyl]-4-bromo-1*H*-indole-2-carboxamide (7)

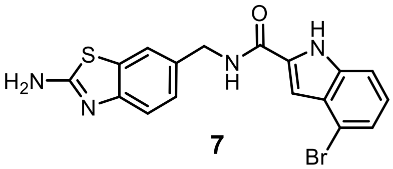

7

6-(Aminomethyl)benzo[*d*]thiazol-2-amine (38 mg, 0.21 mmol), 4-bromoindole-2-carboxylic acid (0.050 g, 0.21 mmol), EDC·HCl (41 mg, 0.21 mmol) and 4-DMAP (25 mg, 0.21 mmol) were dissolved in dry DMF (6 ml) under argon. The resulting light yellow suspension was stirred at r.t. for 23 h after which TLC from the reaction mixture showed formation of product. The solution

was concentrated with the rotary evaporator and the residue was purified by automated column chromatography on silica gel (eluent: EtOAc/*n*-hexane 1:4→1:0) to give **7** as an off-white solid (41 mg, 49%). ^{1}H NMR (400 MHz, DMSO-d_6): δ 11.99 (1H, br s), 9.16 (1H, br t, J = 6.0 Hz), 7.61 (1H, d, J =1.6 Hz), 7.44 (1H, d, J = 8.4 Hz), 7.41 (2H, br s), 7.29 1H, d, J = 8.0 Hz), 7.27 (1H, dd, J = 0.8 Hz, 7.6 Hz), 7.23 (1H, m_c) 7.20 (1 H, dd, J = 1.6 Hz, 8.0 Hz), 7.11 (1H, dd, J = 0.8 Hz, 7.6 Hz), 4.51 (2H, d, J = 6.0 Hz); ^{13}C NMR (100 MHz, DMSO-d_6): δ 166.3, 160.4, 151.9, 136.7, 132.4, 132.0, 131.0, 127.7, 125.2, 124.4, 122.4, 119.8, 117.4, 114.4, 112.0, 102.3, 42.3; LC-MS: $[M+H]^+$, *m/z* 401 (t_r = 2.84 min), ≥95%; HRMS-ESI *m/z*: calc. for $C_{17}H_{13}BrN_4OS$ $[M+H]^+$: 401.0072, found 401.0068.

_N_-[(2-Aminobenzo[_d_]thiazol-6-yl)methyl]-6-bromo-1_H_-indole-2-carboxamide (8)

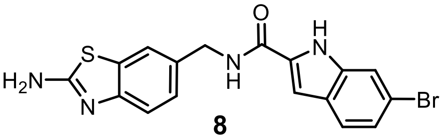

8

6-(Aminomethyl)benzo[*d*]thiazol-2-amine (38 mg, 0.21 mmol), 6-bromoindole-2-carboxylic acid (0.050 g, 0.21 mmol), EDC·HCl (41 mg, 0.21 mmol) and 4-DMAP (25 mg, 0.21 mmol) were dissolved in dry DMF (6 ml) under argon. The resulting light-yellow suspension was stirred at r.t. for 40 h after which TLC from the reaction mixture showed formation of product. The solution was concentrated with the rotary evaporator and the residue was purified by automated column chromatography on silica gel (eluent: EtOAc/*n*-hexane 1:4→1:0) to give **8** as an off-white solid (21 mg, 25%). ^{1}H NMR (400 MHz, DMSO-d_6): δ 11.74 (1 H, br s), 9.06 (1H, br t, J = 6.0 Hz), 7.61–7.58 (3H, m), 7.41 (2H, br s), 7.29 (1H, d, J = 8.4 Hz), 7.20 (1H, m_c) 7.18 (1H, d, J = 8.4 Hz, overlapping), 7.16 (1H, dd, J = 1.6 Hz, 8.4 Hz), 4.51 (2H, d, J = 6.0 Hz); ^{13}C NMR (100 MHz, DMSO-d_6): δ 166.3, 160.6, 151.9, 137.1, 132.5, 132.0, 131.0, 126.1, 125.0, 123.4, 122.7,

119.7, 117.4, 116.0, 114.7, 102.7, 42.2; LC-MS: $[M+H]^+$, *m/z* 401 (t_r = 2.95 min), ≥95%; HRMS-ESI *m/z*: calc. for $C_{17}H_{13}BrN_4OS$ $[M+H]^+$: 401.0072, found 401.0075.

N-[(2-Aminobenzo[d]thiazol-6-yl)methyl]-1H-benz[d]imidazole-2-carboxamide (9)

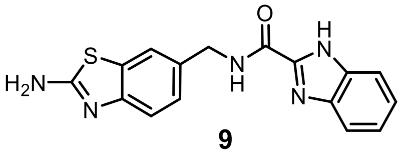

9

6-(Aminomethyl)benzo[*d*]thiazol-2-amine (23 mg, 0.13 mmol), benzimidazole-2-carboxylic acid (21 mg, 0.13 mmol), EDC·HCl (25 mg, 0.13 mmol) and 4-DMAP (16 mg, 0.13 mmol) were dissolved in dry DMF (4 ml) under argon. The resulting light-yellow suspension was stirred at r.t. for 24 h after which TLC from the reaction mixture showed formation of product. The solution was concentrated with the rotary evaporator and the residue was purified by automated column chromatography on silica gel (eluent: EtOAc/*n*-heptane 1:4→1:0) to give **9** as a white solid (12 mg, 29%). ^{1}H NMR (400 MHz, DMF-d_7): δ 13.26 (1H, br s), 9.30 (1H, br t, J = 6.4 Hz), 7.74 (1H, s), 7.70 (2H, br s), 7.50 (2H, br s), 7.35–7.29 (4H, m) 4.67 (2H, d, J = 6.4 Hz); ^{13}C NMR (100 MHz, DMF-d_7): δ 166.9, 159.1, 152.6, 146.2, 143.3, 135.1, 132.5, 131.8, 125.5, 124.2, 122.7, 120.3, 120.0, 117.8, 112.6, 42.6; LC-MS: $[M+H]^+$, *m/z* 324 (t_r = 1.74 min), ≥95%; HRMS-ESI *m/z*: calc. for $C_{16}H_{14}N_5OS$ $[M+H]^+$: 324.0919, found 324.0919.

Supplementary Figures

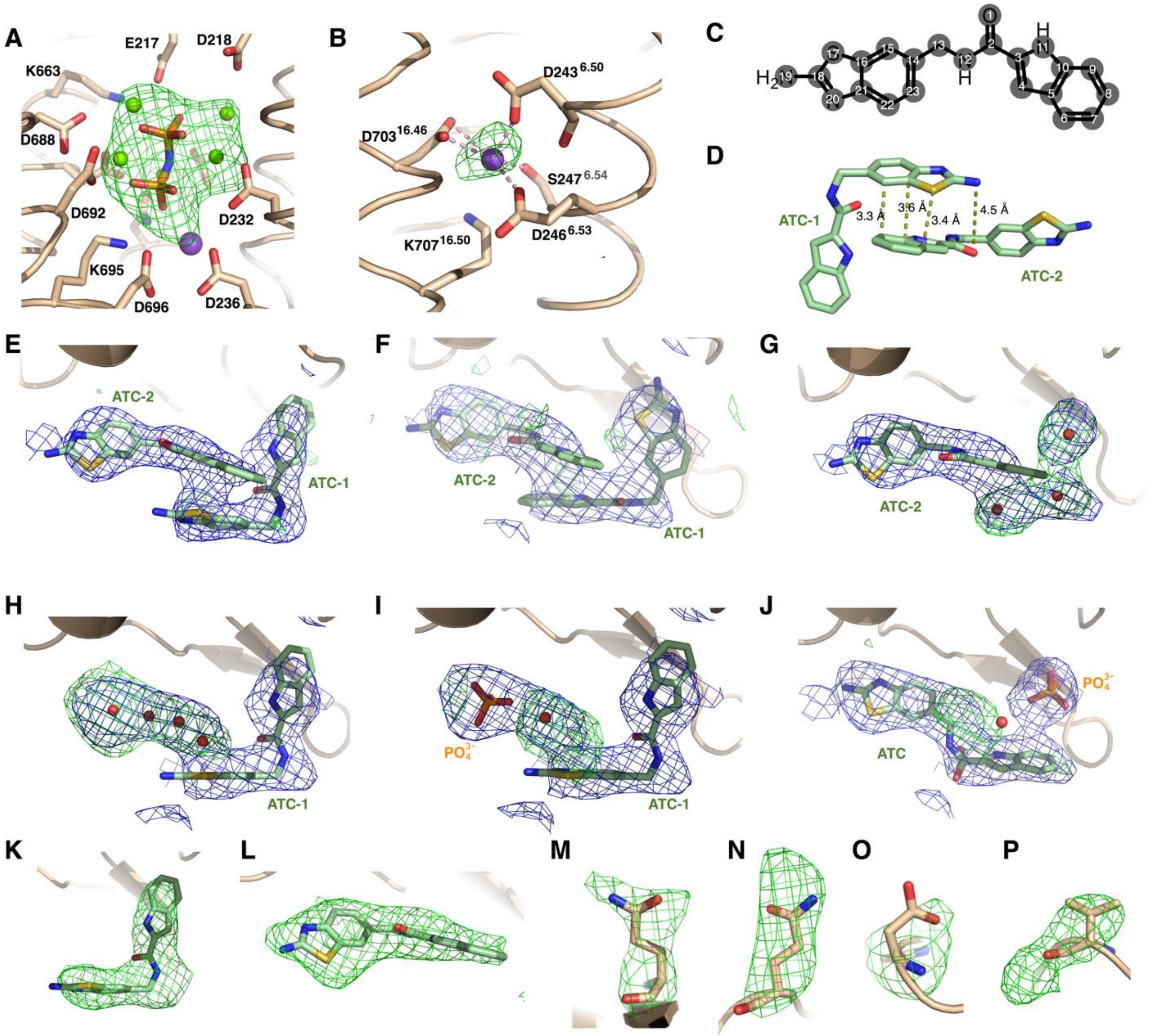

Fig. S1. Electron density maps of ligands in the TmPPase:IDP:ATC structure. (**A**) Omit (F_o–F_c) map contoured at 3σ (green mesh) in the hydrolytic center, into which five magnesium ions (green spheres), a potassium ion (violet-purple sphere) and IDP can be fit. (**B**) Omit (F_o–F_c) map contoured at 3σ (green mesh) electron density map at the gate, which can be fitted with a sodium ion (violet-purple sphere). The sodium ion is coordinated by oxygens from three Asp residues and one serine. (**C**) Numbering scheme of the atoms in ATC. (**D**) Distances between dimer interface of ATC-**1** and ATC-**2**. (**E**) ($2F_o$–F_c) (blue mesh) and (F_o–F_c) (green mesh) electron density map of ATC-**1** and ATC-**2** at 1σ and 3σ, respectively. (**F–J**) Electron density maps as in D, but with different molecules built into the density and refined: (**F**): ATC-**1** orientation reversed; (**G**) ATC-

1 replaced with three water molecules (red dots); (**H**) ATC-**2** replaced with four water molecules (red dots); (**I**) ATC-**2** replaced with a water molecule (red dot) and phosphate ion (orange); (**J**) ATC in a different position with a water molecule (red dot) and a phosphate ion (orange). In all the maps (**F–J**), there is considerable positive (F_o–F_c) electron density; the only model that fits as well as our preferred model has a reverse ATC-1. (**K–P**) Polder OMIT maps of ATC-**1**, ATC-**2**, Q268, Q277, D351 and V352, demonstrating that the ATC-**1** & ATC-**2** fit the electron density similarly to the protein side chains.

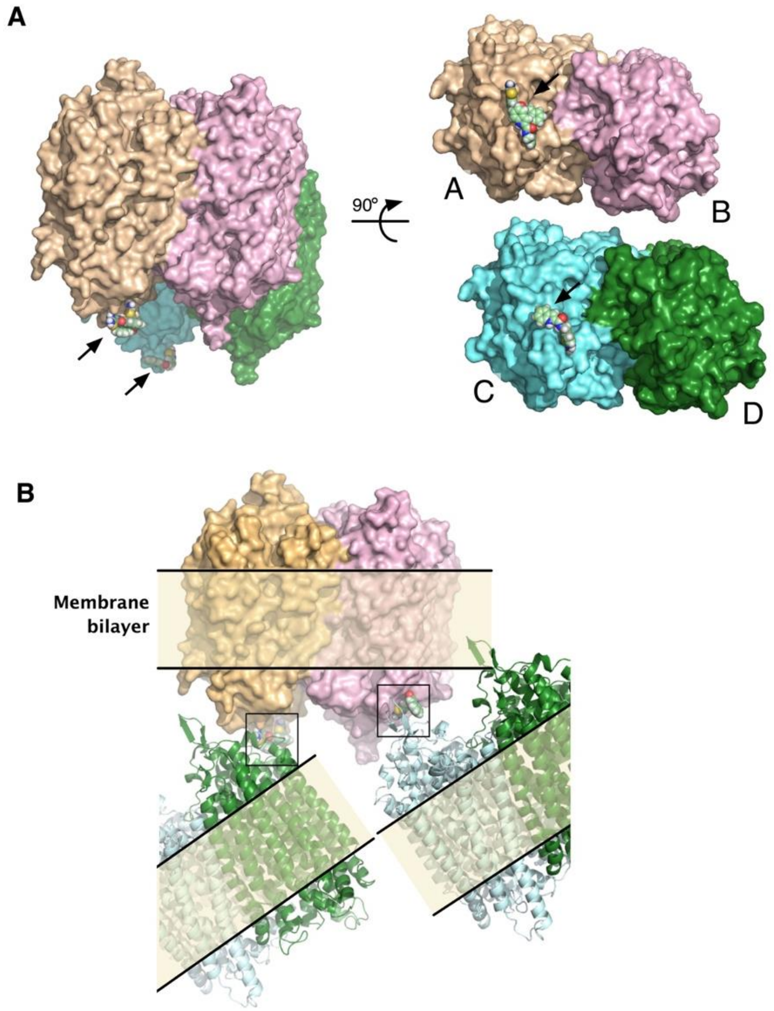

Fig. S2. Asymmetric unit of TmPPase:IDP:ATC. (**A**) Four TmPPase chains in the asymmetric unit showing the dimer arrangement and ATC binding site (arrow). Color scheme: chain A, wheat; chain B, light pink; chain C, cyan; chain D, forest green. (**B**) Interaction between chain AB with CD via ATC in the crystal contact. The small boxes indicate the ATC binding sites.

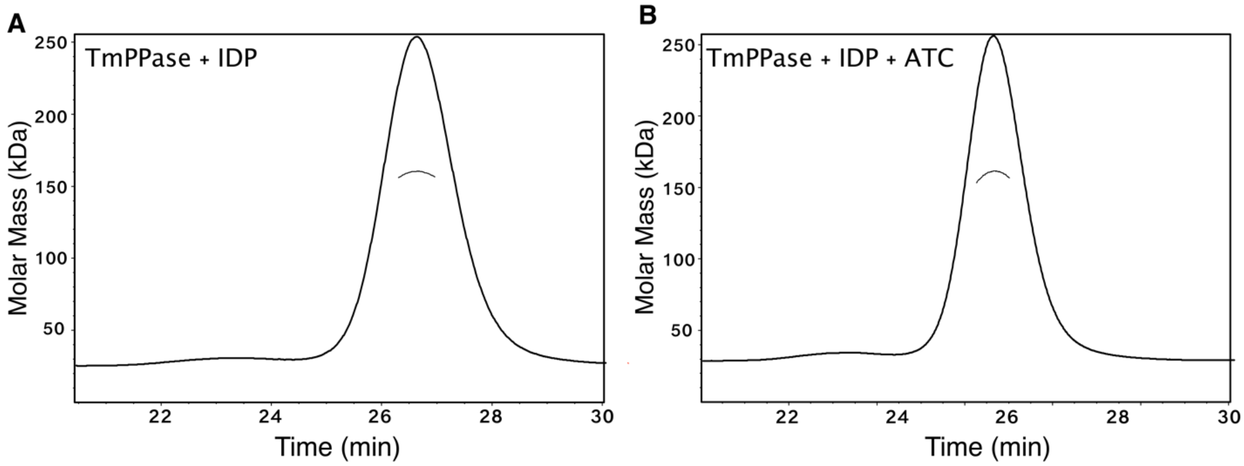

Fig. S3. Oligomerization state of TmPPase upon the addition of inhibitors. Size-exclusion chromatography – multiangle laser light scattering (SEC-MALLS) analysis of (**A**) TmPPase+IDP and (**B**) TmPPase+IDP+ATC. Adding ATC to the protein-IDP complex did not change the oligomerization state. Line across the light scattering chromatogram peak shows the molecular weight distribution of the protein.

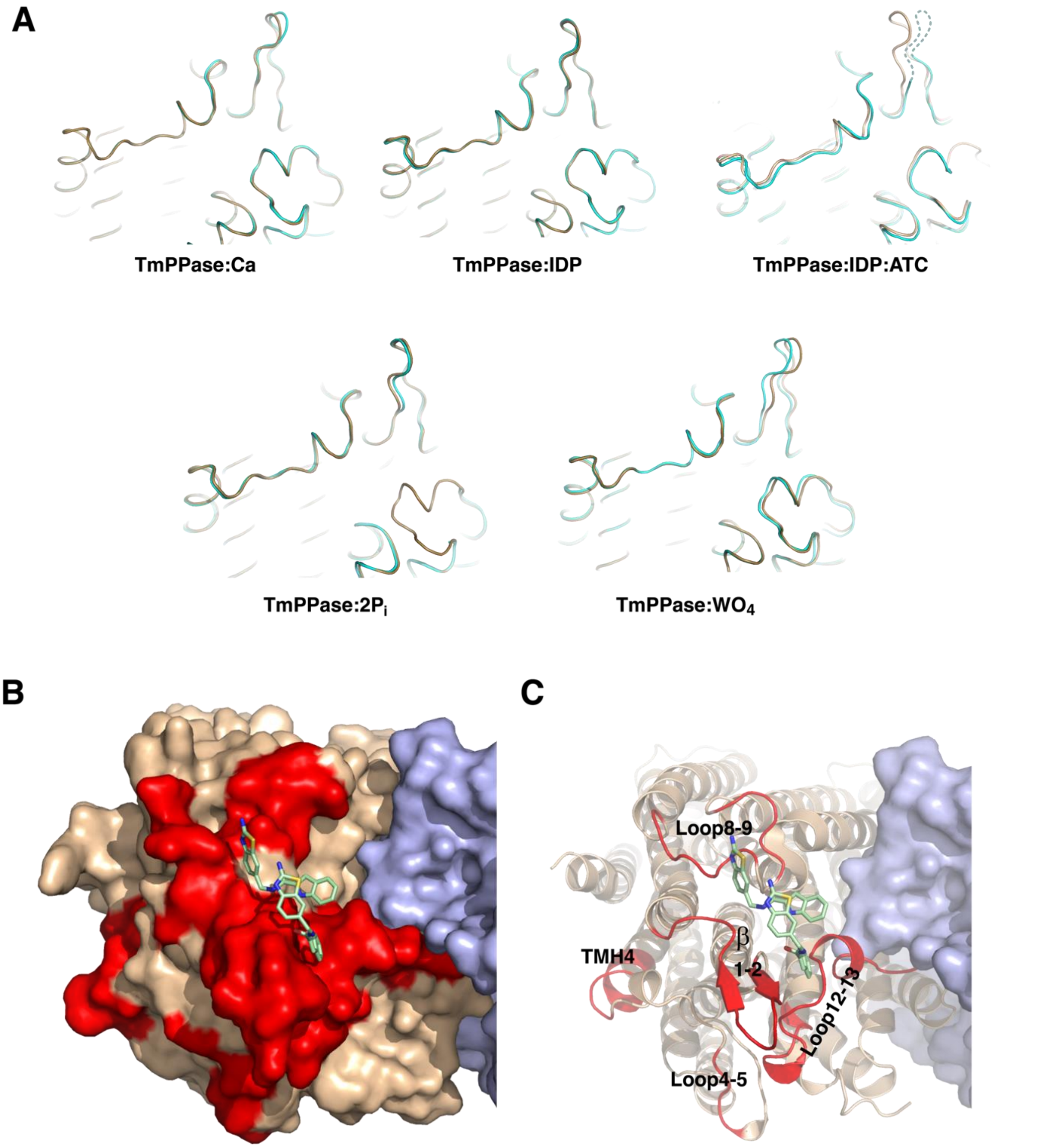

Fig. S4. Comparison of the ATC binding sites in different TmPPase structures. (**A**) Superposition of ATC binding site between chain A (wheat) and B (cyan) of different TmPPase states. (**B**) Surface and (**C**) cartoon representation of region near exit channel showing the mobile region (red) around the ATC binding site.

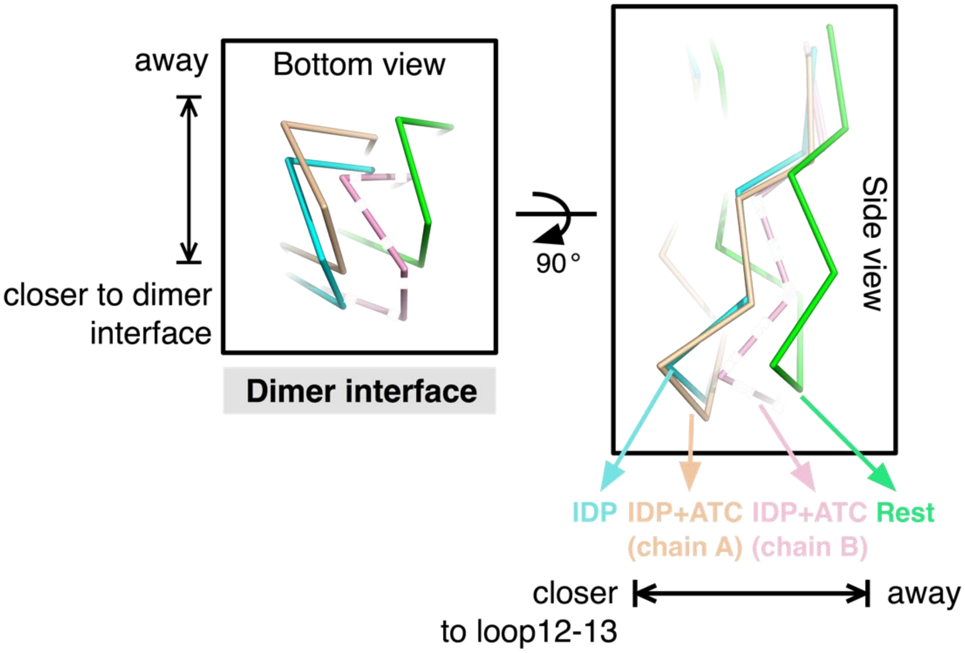

Fig. S5. Differences in the ATC binding site between monomers of the TmPPase:IDP:ATC structure. Superposition of chain A and B of the IDP:ATC-bound state in comparison with chain A of the resting state and IDP-bound state mPPase showing the closer/away movement of β1–2 strand relative to the loop12–13 and the dimer interface of the protein. β1–2 strand of chain B is represented with dashed ribbon as this region is flexible and not visible in the structure. Coloring scheme: resting state (TmPPase:Ca:Mg) = green; chain B of IDP:ATC-bound state = light pink; chain A of IDP:ATC-bound state = wheat; IDP-bound state (TmPPase:IDP)= cyan, ATC = pale green.

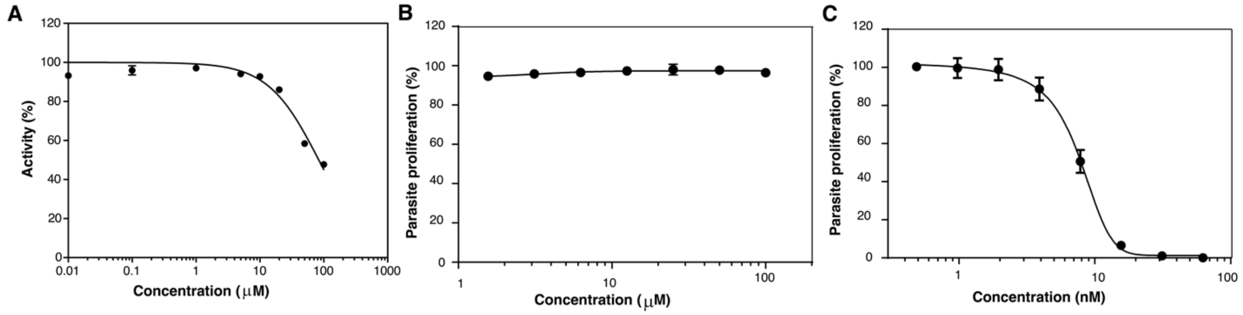

Fig. S6. Effect of ATC on growth of *P. falciparum* and on PfPPase. (**A**) Inhibition effect of ATC on PfPPase-VP1 (in membrane) from *P. falciparum* expressed in insect cell baculovirus expression system (method and further analysis will be described in more detail elsewhere). ATC inhibits the protein at $\log IC_{50}$ of 1.9 ± 0.03 (IC_{50} = 83.6 μM). (**B**) ATC has no anti-plasmodial activity up to 100 μM. (**C**) Anti-plasmodial activity of artemisinin, as a positive control.

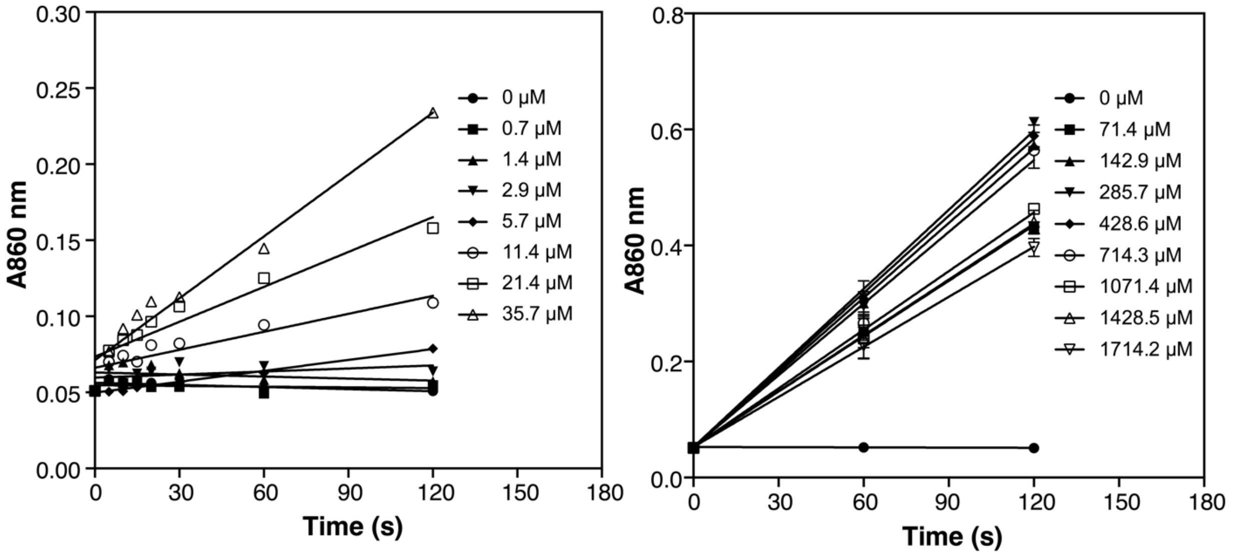

Fig. S7. Linearity of TmPPase activity as a function of time and concentration. Multiple time points were measured for all of the PP_i concentrations used (left panel: 0–35.7 μM; right panel: 71.4–1714.2 μM). At two minutes reaction time, the absorbance at 860 nm is still in the linear range, so two minutes was used in the kinetic analysis (Fig. 4).

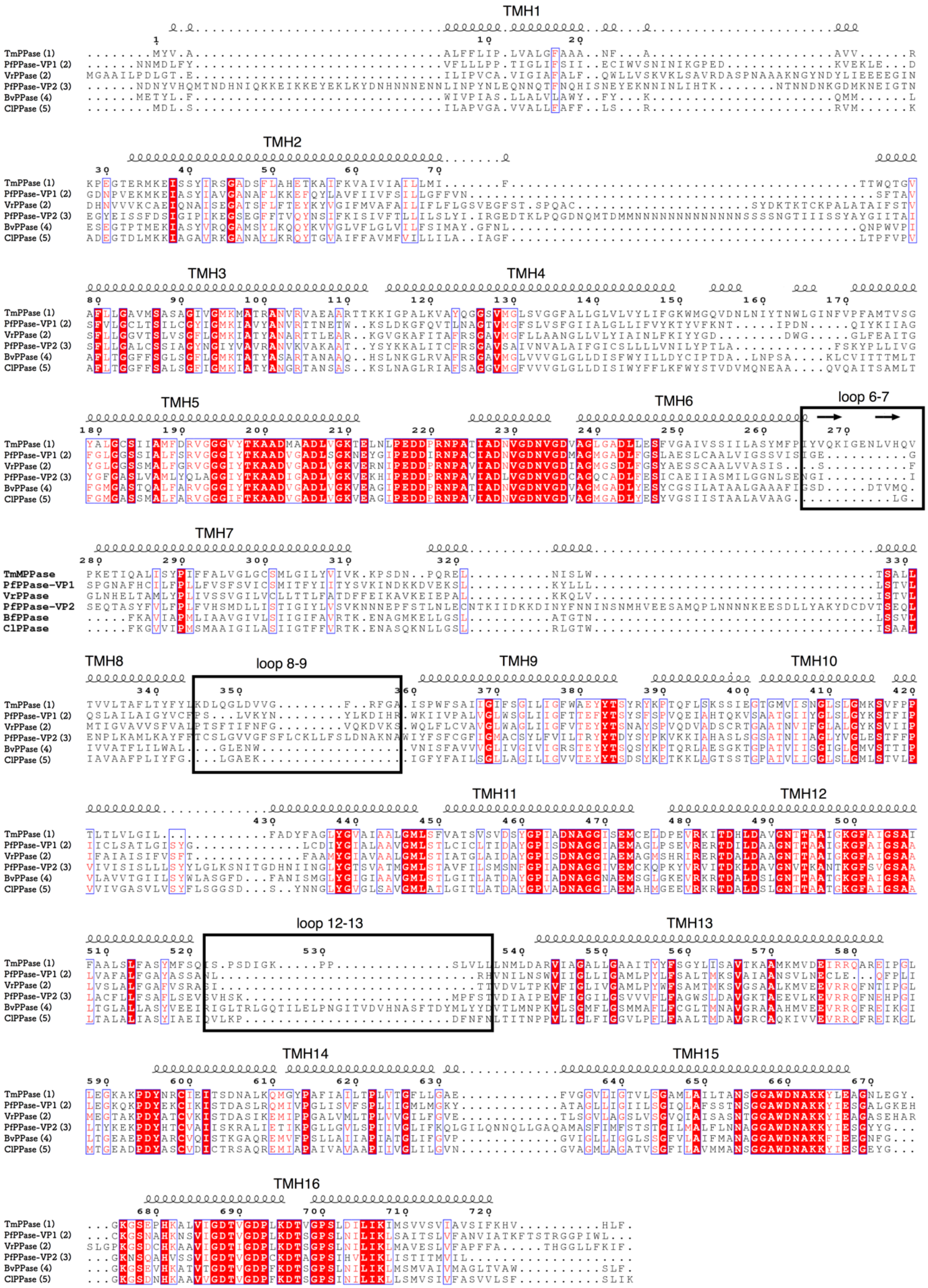
TMH1
TmPPase (1)
PfPPase-VP1 (2)
VrPPase (2)
PfPPase-VP2 (3)
BvPPase (4)
ClPPase (5)
TMH2
TMH3
TMH4
TMH5
TMH6
loop 6-7
TMH7
TmMPPase
PfPPase-VP1
VrPPase
PfPPase-VP2
BfPPase
ClPPase
TMH8
loop 8-9
TMH9
TMH10
TMH11
TMH12
loop 12-13
TMH13
TMH14
TMH15
TMH16

Fig. S8. Multiple sequence alignment of mPPases from different organisms. We only show PfPPase-VP1 and PfPPase-VP2 as representative of the 16 parasite mPPases, all of which have 40–70% sequence identity to each other, and none of which have loop 6-7. Numbering after mPPase corresponds to subfamilies: (1): Na^+-pumping K^+-dependent (TmPPase from *T. maritima*); (2): H^+-pumping K^+-dependent (PfPPase-VP1 from *P. falciparum* and VrPPase from *V. radiata*); (3): H^+-pumping K^+-independent (PfPPase-VP2 from *P. falciparum*); (4): Na^+,H^+-pumping mPPase (BvPPase from *Bacteroides vulgatus*); and (5): Na^+-regulated Na^+,H^+-pumping mPPase (ClPPase from *Clostridium leptum*)). Secondary structure is based on the structure of TmPPase. Black rounded rectangles show loops important for ATC binding. Numbering above the sequence corresponds to the residue number in TmPPase. Red box and white characters: identical residues; blue frame and red characters: similar residues. The figure was generated with ESPript 3.0 (http://espript.ibcp.fr/ESPript/ESPript/index.php).

Supplementary Schemes

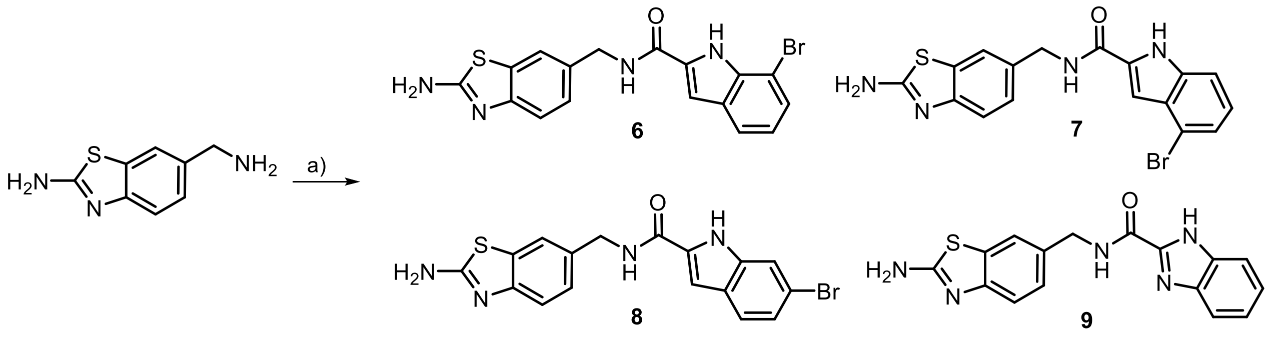

Scheme S1. Synthesis of analogs of 1 with heavy and basic atoms. a) Reagents and conditions: Br-indole-2-carboxylic acid/ benzimidazole-2-carboxylic acid (1.0 eq.), EDC HCl (1.0 eq.), 4-DMAP (1.0 eq.), DMF, RT, 23-40 h (*38*).

Supplementary Tables

Table S1. X-ray data collection and refinement statistics.

**Data Collection**	
Space group	P $2_12_12_1$
Cell dimensions	
a, b, c (Å)	98.1, 141.6, 252.1
α, β, γ (°)	90, 90, 90
Source	ESRF - ID29
Wavelength (Å)	0.9677
Resolution (Å)	47.49 – 3.42 (3.73 – 3.42)
Anisotropy direction*	
Resolution where $CC_{1/2}$>0.50	
Overall (Å)	3.42
along h axis	3.57
along k axis	4.11
along l axis	3.41
Measured reflections	186550 (7994)
Unique reflections	36342 (1846)
Completeness (%)	93.7 (57.4)
$CC_{1/2}$	0.99 (0.56)
Mean $I/\sigma(I)$	9.2 (1.5)
Multiplicity	5.1 (4.3)
Wilson B ($Å^2$)	98.7
R_{merge}	0.11 (0.98)
R_{meas}	0.13 (1.11)
R_{pim}	0.06 (0.52)

**Refinement**	
Resolution (Å)	47.5 – 3.4
R_{work}(%)/R_{free}(%)	22.8/28.4
No. of atoms	21042
protein	20895
ligands	139
water	8
No. of chains (ASU)	4
B-factors ($Å^2$)	86.1
Protein	86.0
Ligands/Ion	100.5
R.M.S. Deviations	
Bond lengths (Å)	0.002
Bond angle (°)	0.44
Ramachandran statistics†	
Favored (%)	95.71
Allowed (%)	4.29
Outliers (%)	0.00

Statistics for the highest-resolution shell are shown in parentheses.
*The anisotropy statistics were computed with AIMLESS after use of the StarAniso server.

†Ramachandran statistics were calculated with MolProbity.

$CC_{1/2}$, correlation coefficient; h, k, l: indices that define the lattice planes, $I/\sigma(I)$, empirical signal-to-noise ratio; R_{merge}, quality agreement among equivalent reflections to asses X-ray data quality; R_{meas}, multiplicity-corrected R; R_{pim}, expected precision.

Table S2. RMSD of TmPPase monomer in the asymmetric unit relative to monomer A.

**Monomer**	**RMSD ($Å^2$)**
B	0.31
C	0.26
D	0.33

Table S3. Major interactions of ATC with chains A and D.

Interaction type	Compound	Chain A		Chain D	
		**Residue**	**BSA* (Å^2)**	**Residue**	**BSA* (Å^2)**
Hydrophobic/<br>Aromatic	ATC-1	Q268<br>K269<br>I270<br>P530	42.1<br>23.4<br>22.7<br>24.2	I70<br>T73<br>W74<br>W164	18.8<br>19.4<br>20.0<br>26.4
	ATC-2	Q268<br>Q277<br>D351<br>V352	32.3<br>23.8<br>24.7<br>18.8	W74<br>Y161<br>F169	41.0<br>32.4<br>18.3
	Total BSA* (Å^2)		212.0		176.3
Possible hydrogen bond	**Compound**	**Chain A**	**Distance (Å)**	**Chain D**	**Distance (Å)**
	ATC-1 (O01)	Q268 (Nε2)	2.7		
	ATC-2 (N11)<br>ATC-2 (N12)<br>ATC-2 (N19)	Q268 (Oε1)<br>Q268 (Oε1)	3.0<br>3.0	N157 (Nδ2)	3.1
Salt bridge	ATC-2 (N20)	D351 (Oδ1)	3.4		

* BSA: Buried surface area: the solvent-accessible surface area (cut-off = 15 Å^2) of the corresponding residue that is buried upon interface formation.

Table S4. RMSD between chains A and B of loops in different TmPPase structures.

**Loop** (residues)	**RMSD** (Å)[1]				
	TmPPase:IDP:ATC	5LZQ	5LZR	4AV3	4AV6
2-3 (71-75)	0.63	0.18	0.44	0.33	0.26
4-5 (153-172)	1.11	0.24	0.58	0.37	0.32
β1-2 (6-7) (266-276)	1.30[2]	0.49	1.24	0.61	0.50
8-9 (342-359)	0.79[3]	0.41	0.70	0.34	0.35[4]
12-13 (520-540)	0.80	0.37	0.41	0.46	0.36

[1] Comparisons were done by superimposing helix 7 (aa. 281-311), which moves least between the structures.
[2] Missing residues 269-274. RMSD was measured between the remaining residues.
[3] Missing residues 346-349. RMSD was measured between the remaining residues.
[4] Missing residues 349-357. RMSD was measured between the remaining residues.

Table S5. **RMSD of chain A of TmPPase:IDP:ATC loops to chain A of TmPPase:IDP loops.**

**Loop** (residues)	**RMSD** (Å)[1]
	Monomer A
2-3 (71-75)	0.81
4-5 (153-172)	0.75
β1-2 (6-7) (266-276)	1.13
8-9 (342-359)	0.70
12-13 (520-540)	1.09

[1] Comparisons were made by superimposing helix 7 (residues 281-311), which moves least between the TmPPase:IDP and TmPPase:IDP:ATC structures

Table S6. Hill constant of TmPPase inhibition by ATC at different substrate concentrations.

**PP_i conc.** (μM)	**Hill constant**
35	2.8 ± 0.28
71	2.8 ± 0.22
142	1.9 ± 0.17
285	2.2 ± 0.14
1071	2.2 ± 0.18
1428	1.9 ± 0.21
1721	2.0 ± 0.21

^{1}H NMR and ^{13}C NMR Spectra

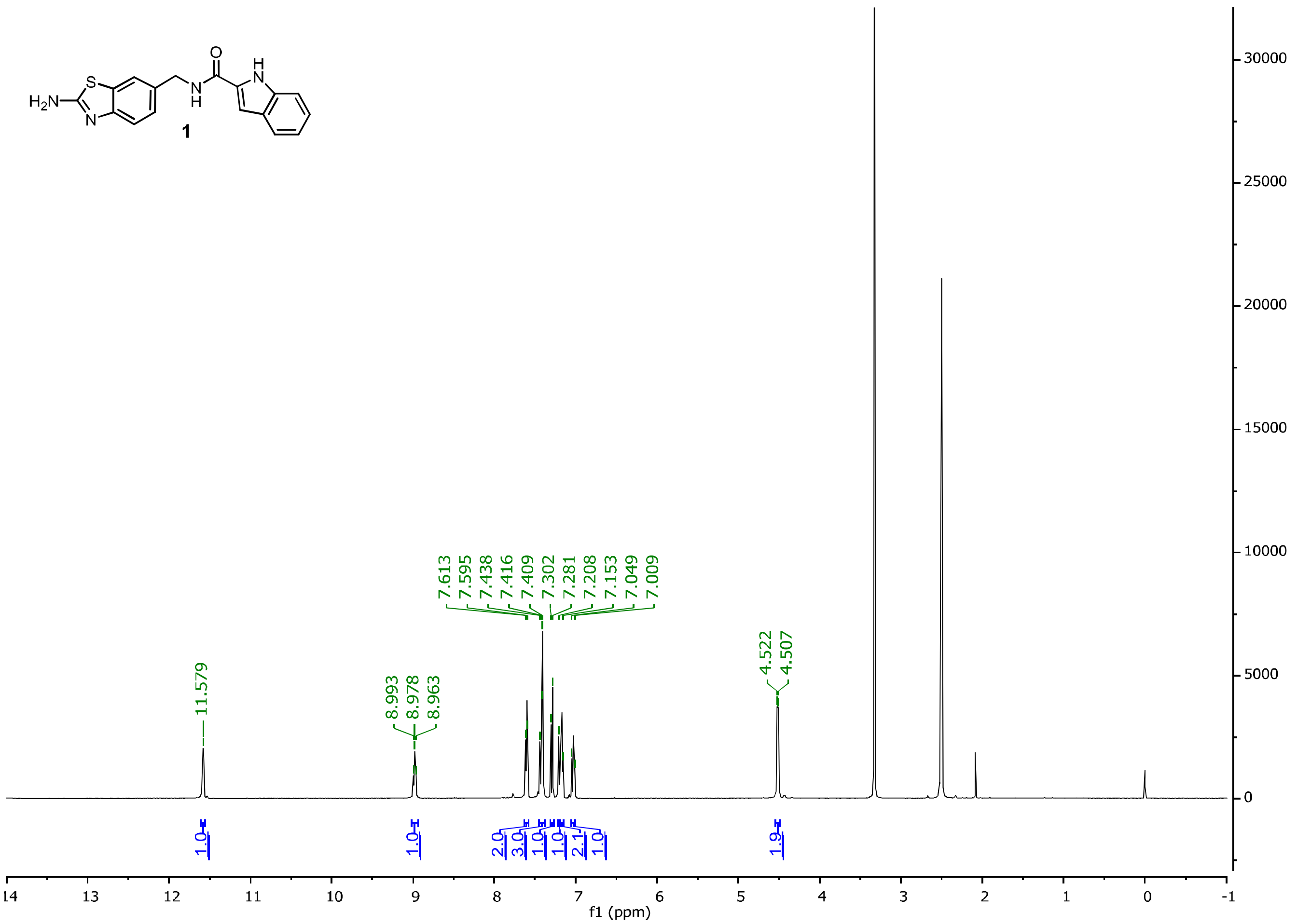

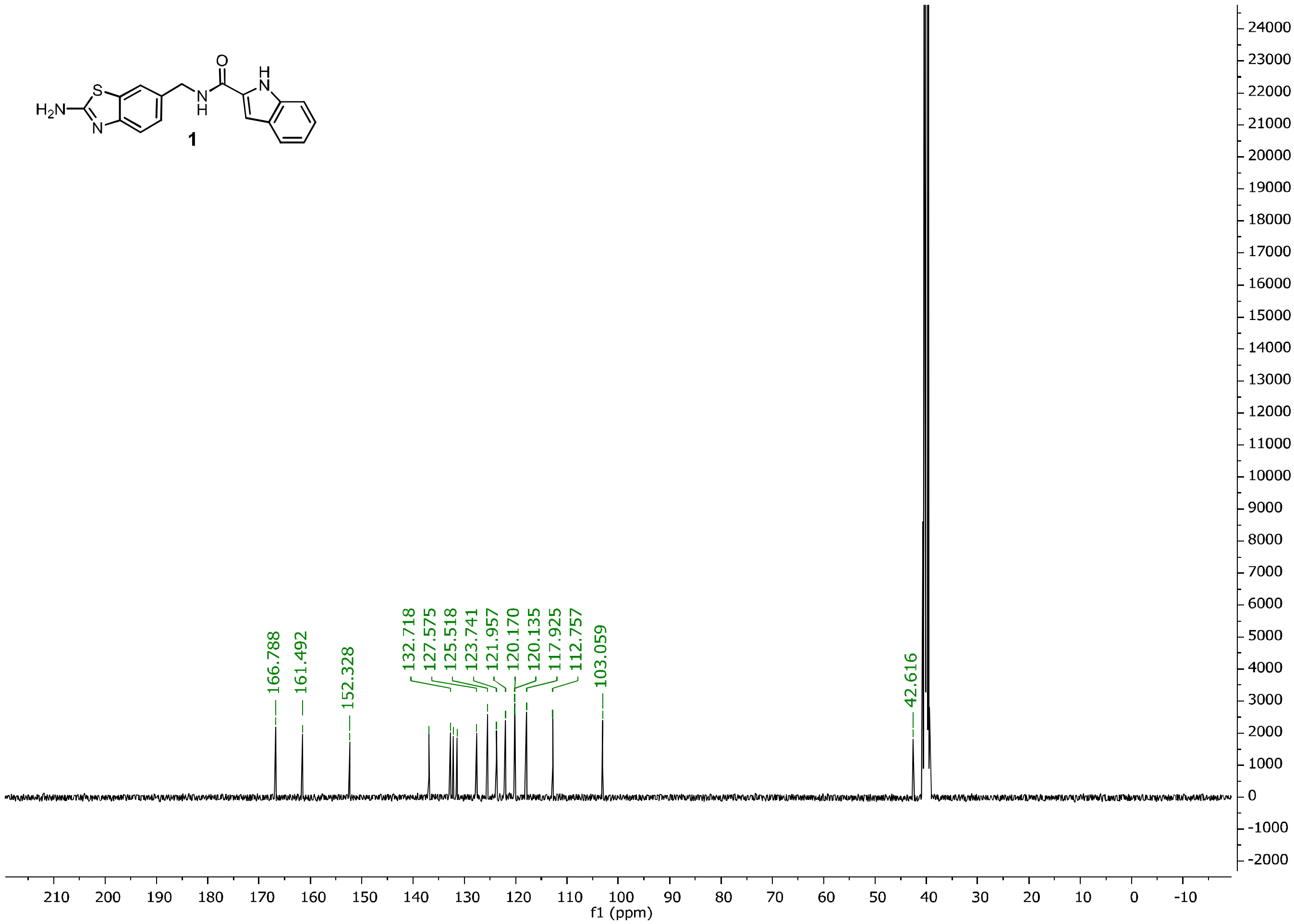
1
166.788
161.492
152.328
132.718
127.575
125.518
123.741
121.957
120.170
120.135
117.925
112.757
103.059
42.616
f1 (ppm)

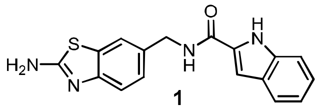

H2N
S
N
N
H
O
H
N

1

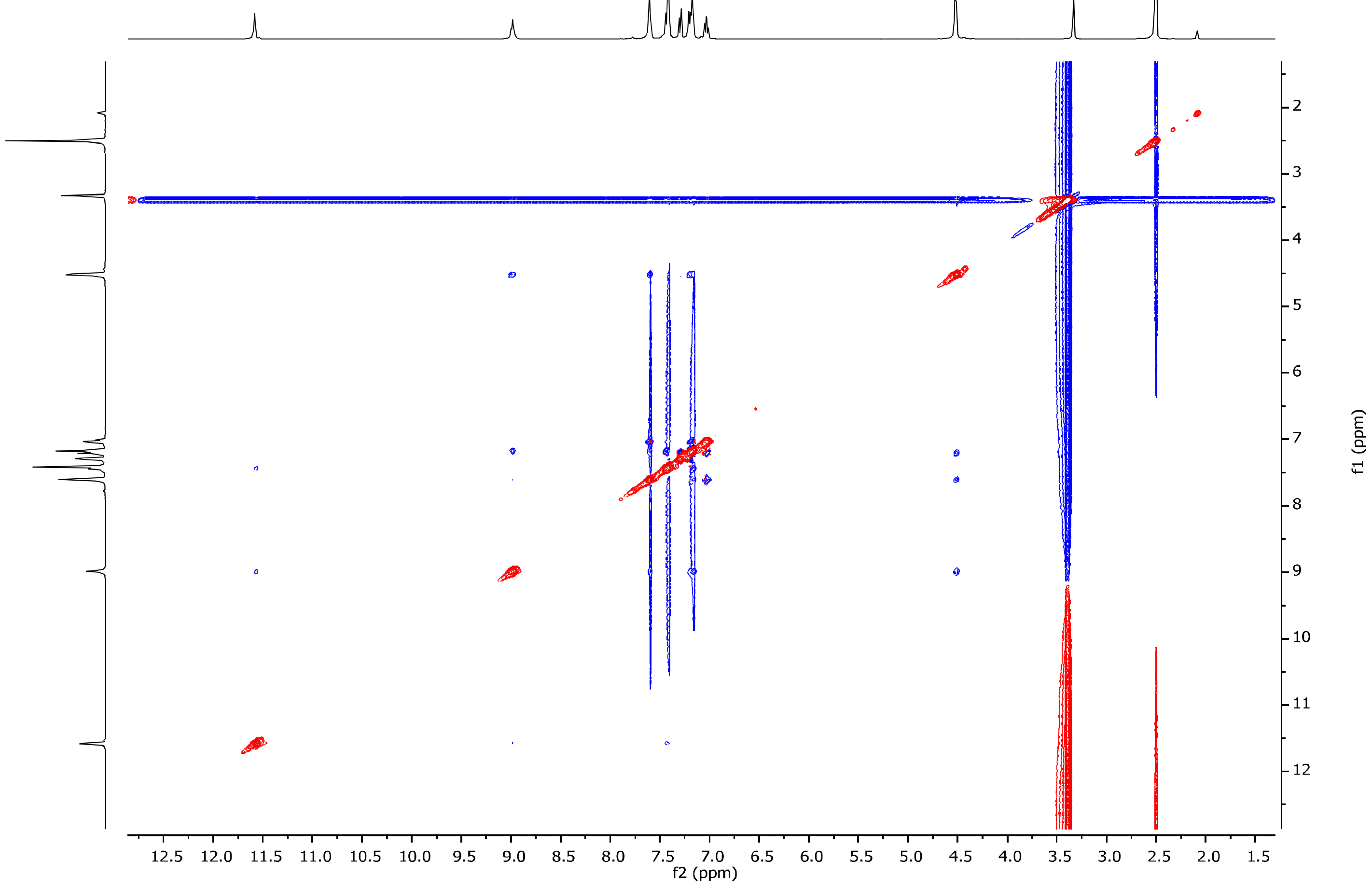

f1 (ppm)
2
3
4
5
6
7
8
9
10
11
12
12.5 12.0 11.5 11.0 10.5 10.0 9.5 9.0 8.5 8.0 7.5 7.0 6.5 6.0 5.5 5.0 4.5 4.0 3.5 3.0 2.5 2.0 1.5
f2 (ppm)

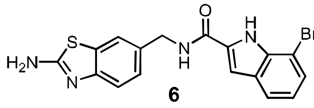

6

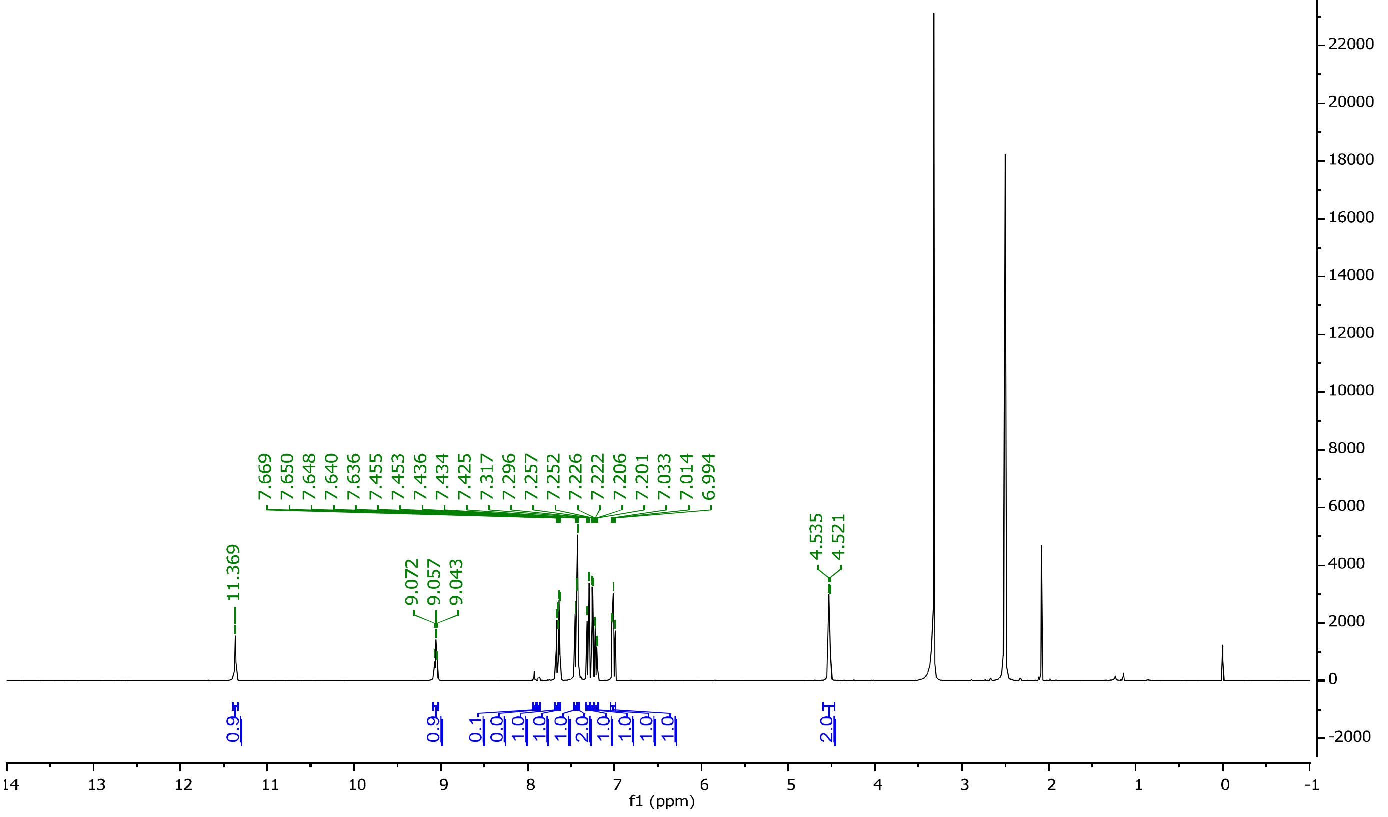

11.369
9.072
9.057
9.043
7.669
7.650
7.648
7.640
7.636
7.455
7.453
7.436
7.434
7.425
7.317
7.296
7.257
7.252
7.226
7.222
7.206
7.201
7.033
7.014
6.994
4.535
4.521
0.9
0.9
0.1
0.0
1.0
1.0
1.0
2.0
1.0
1.0
1.0
1.0
2.0
f1 (ppm)

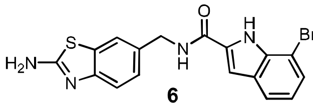
H2N
S
N
O
H
N
Br

6

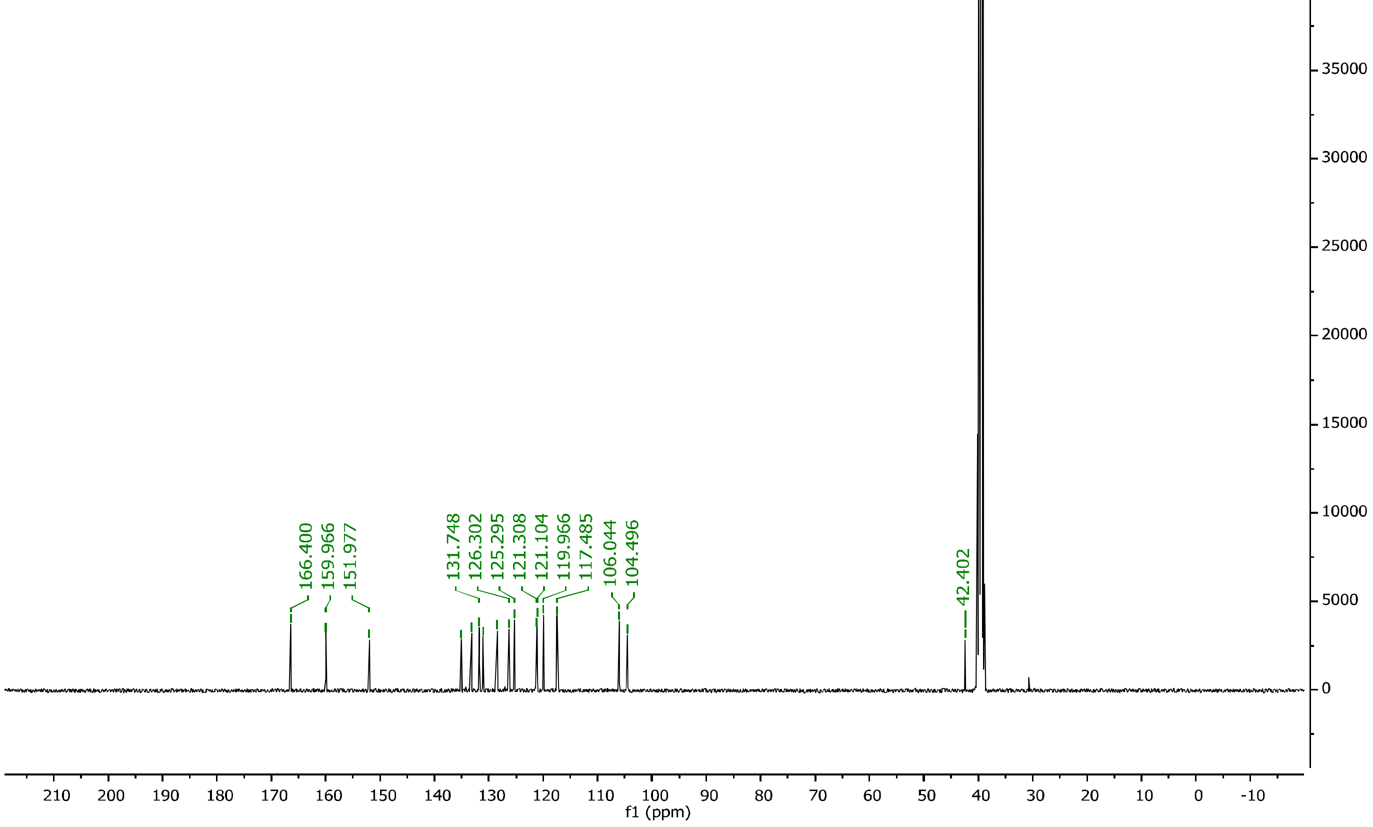
166.400
159.966
151.977
131.748
126.302
125.295
121.308
121.104
119.966
117.485
106.044
104.496
42.402
45000
40000
35000
30000
25000
20000
15000
10000
5000
0
210 200 190 180 170 160 150 140 130 120 110 100 90 80 70 60 50 40 30 20 10 0 -10
f1 (ppm)

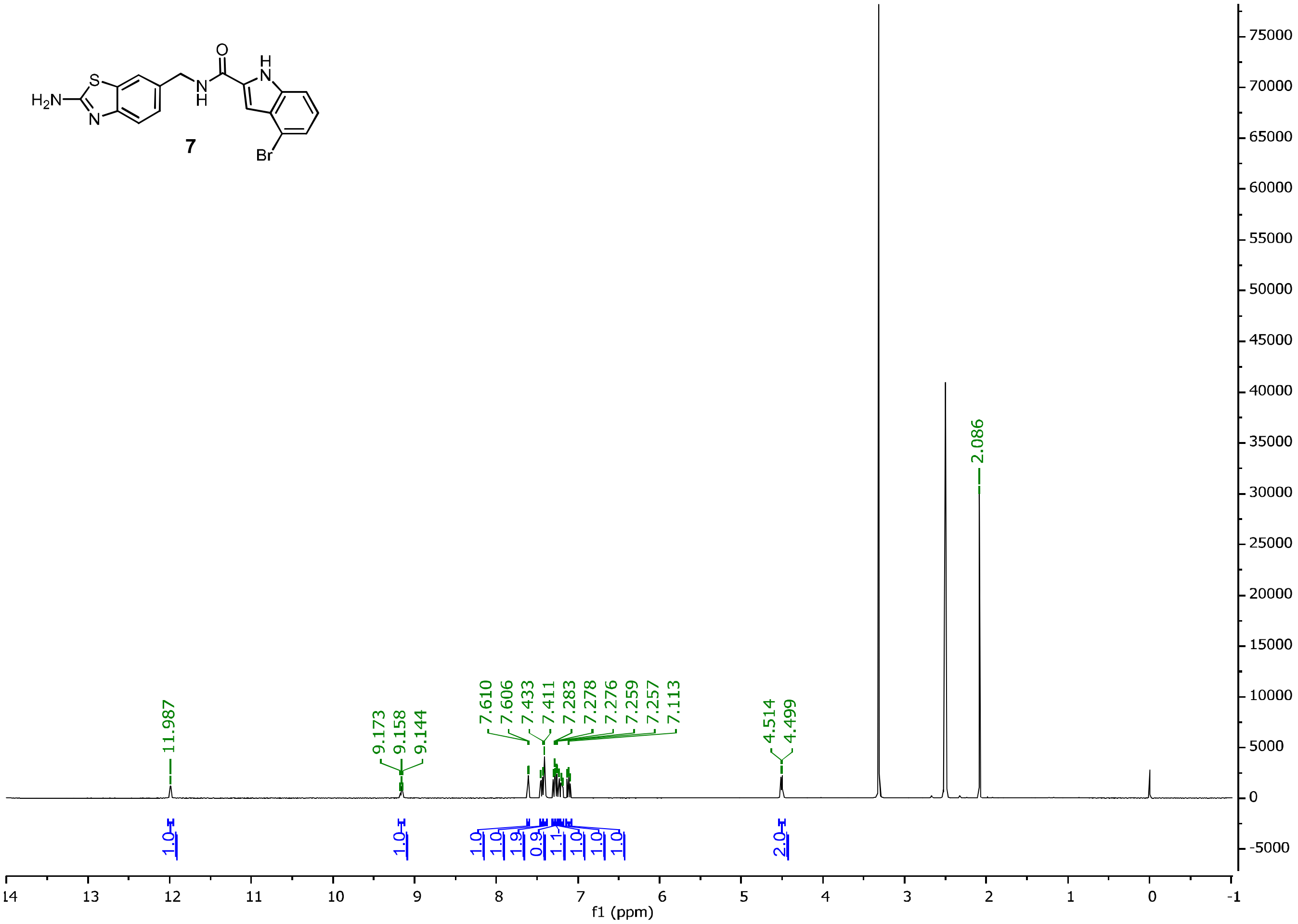
H2N
S
N
N
H
O
H
N
Br
7
11.987
9.173
9.158
9.144
7.610
7.606
7.433
7.411
7.283
7.278
7.276
7.259
7.257
7.113
4.514
4.499
2.086
1.0
1.0
1.0
1.0
1.0
0.9
1.1
1.0
1.0
1.0
2.0
75000
70000
65000
60000
55000
50000
45000
40000
35000
30000
25000
20000
15000
10000
5000
0
-5000
14
13
12
11
10
9
8
7
6
5
4
3
2
1
0
-1
f1 (ppm)

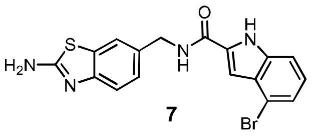
H2N
S
N
O
H
N
N
H
Br

7

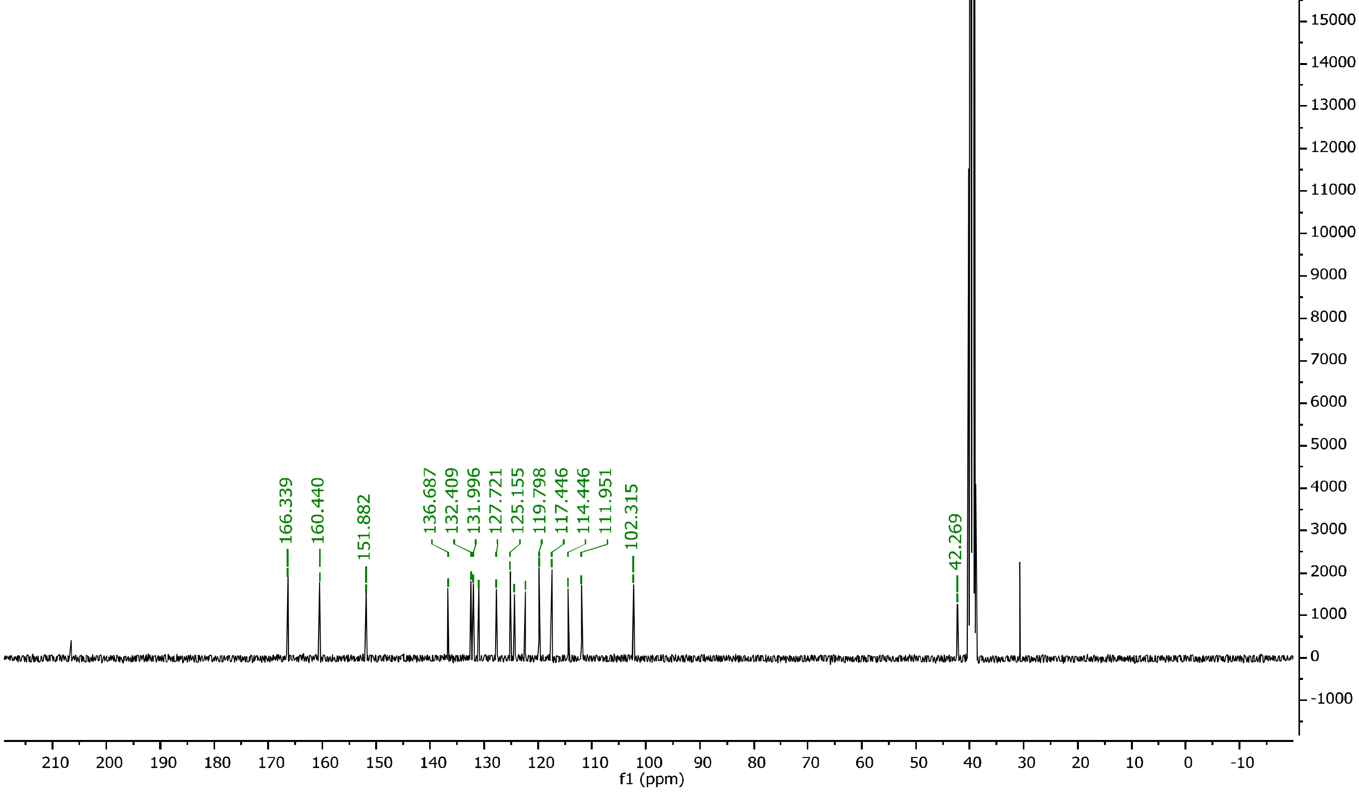
166.339
160.440
151.882
136.687
132.409
131.996
127.721
125.155
119.798
117.446
114.446
111.951
102.315
42.269
f1 (ppm)

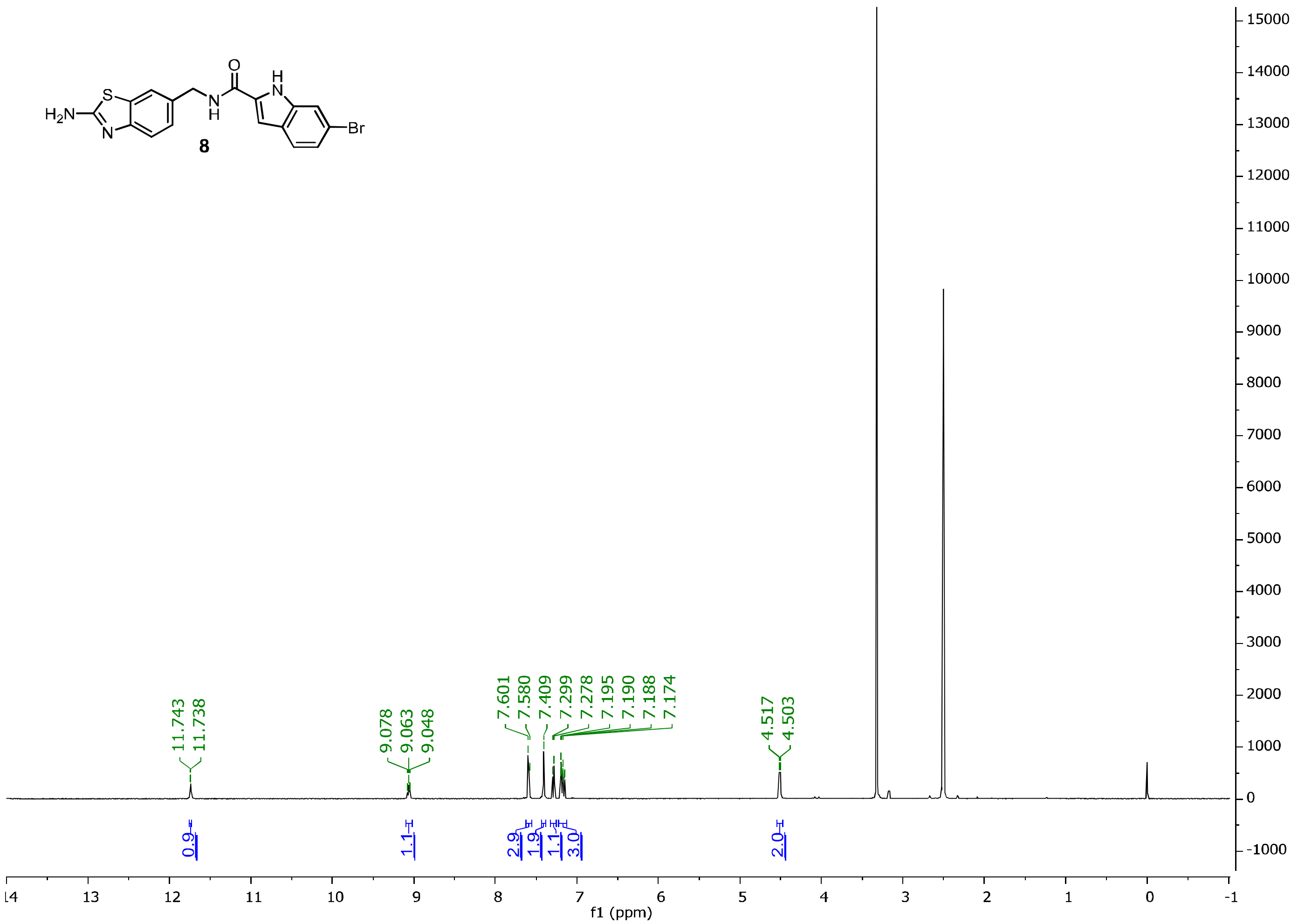

H2N
S
N
O
H
N
N
H
Br
8
11.743
11.738
9.078
9.063
9.048
7.601
7.580
7.409
7.299
7.278
7.195
7.190
7.188
7.174
4.517
4.503
0.9
1.1
2.9
1.9
1.1
3.0
2.0
f1 (ppm)

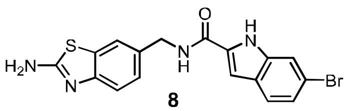

8

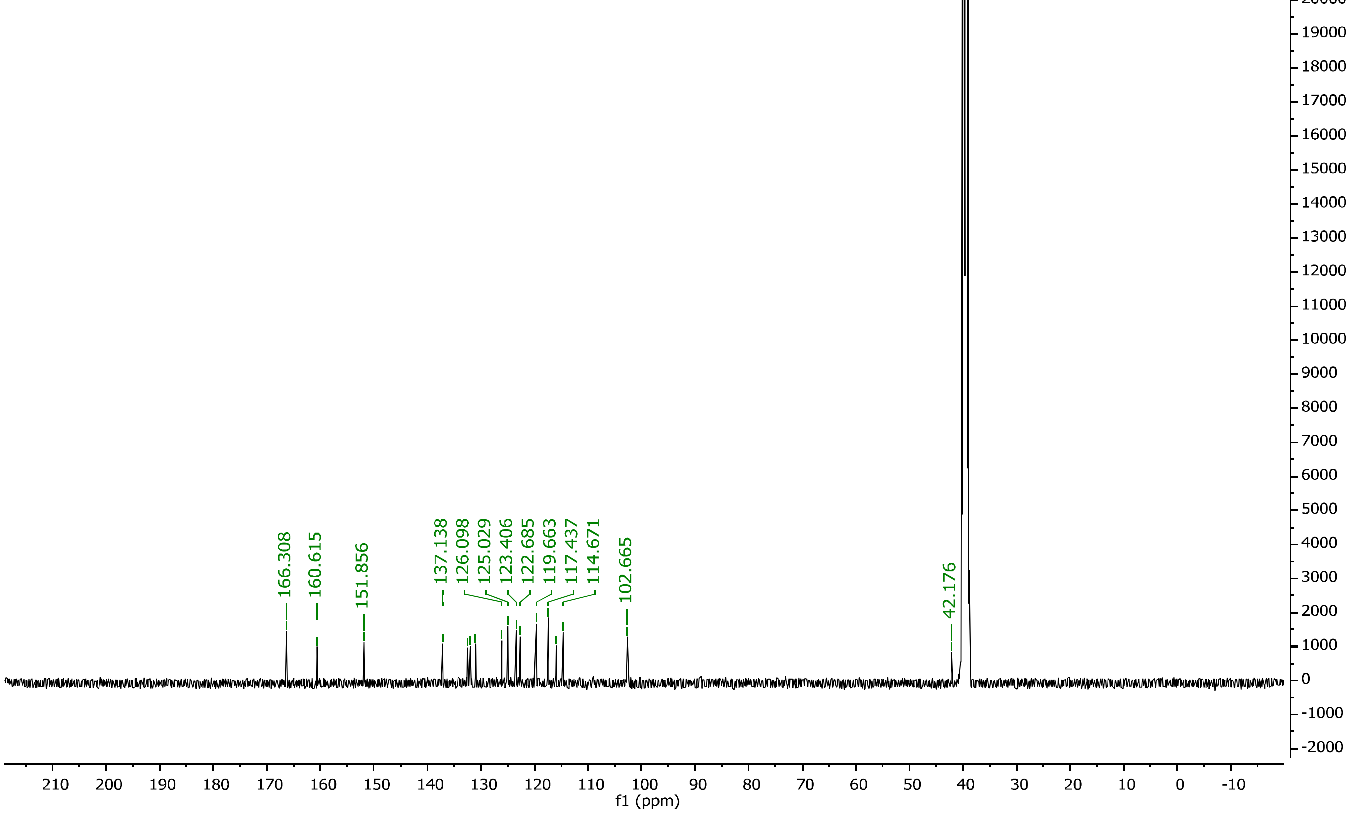

166.308
160.615
151.856
137.138
126.098
125.029
123.406
122.685
119.663
117.437
114.671
102.665
42.176
f1 (ppm)

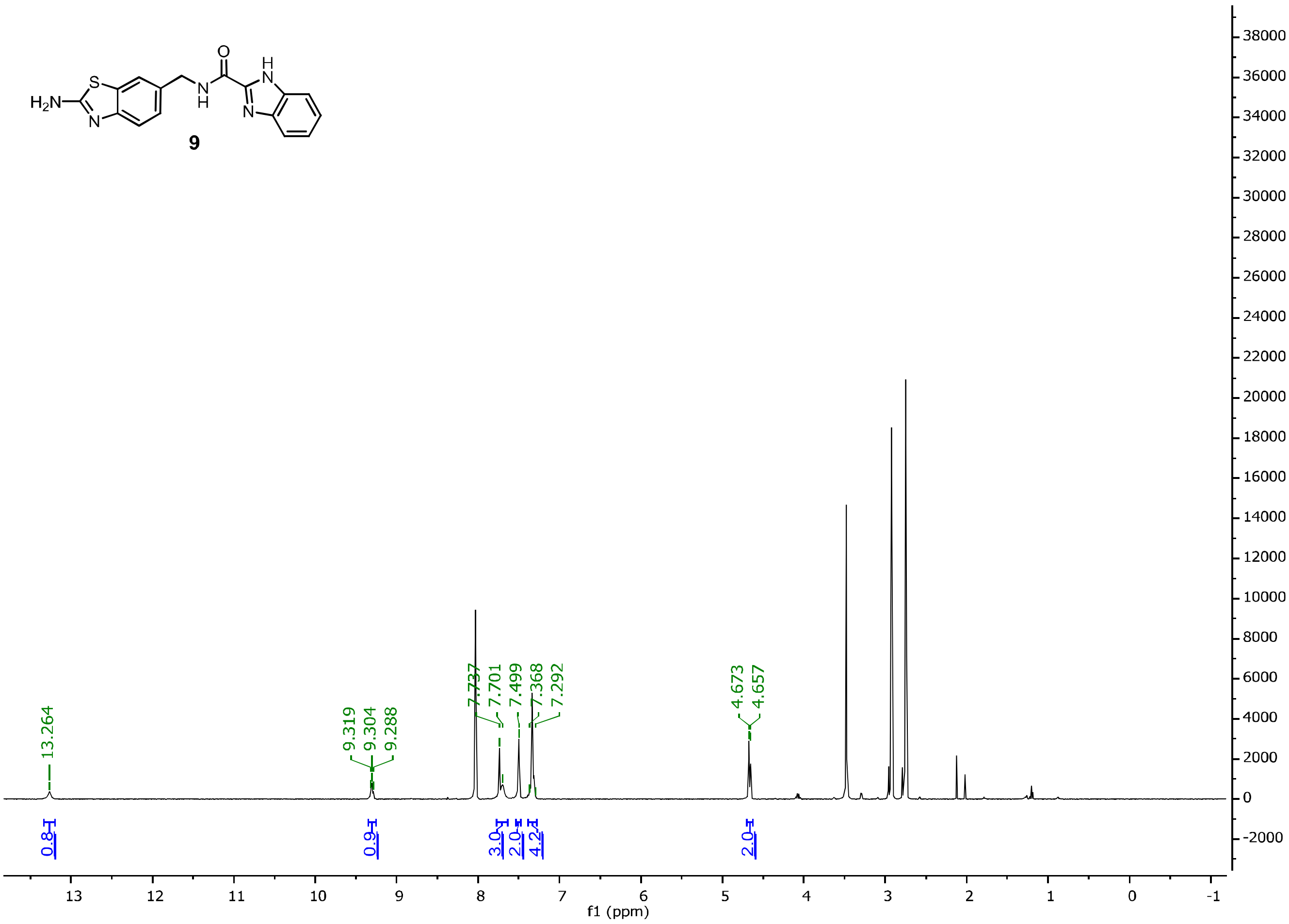
H2N
S
N
O
N
H
H
N
N
9
13.264
9.319
9.304
9.288
7.737
7.701
7.499
7.368
7.292
4.673
4.657
0.8
0.9
3.0
2.0
4.2
2.0
f1 (ppm)
13 12 11 10 9 8 7 6 5 4 3 2 1 0 -1
38000 36000 34000 32000 30000 28000 26000 24000 22000 20000 18000 16000 14000 12000 10000 8000 6000 4000 2000 0 -2000

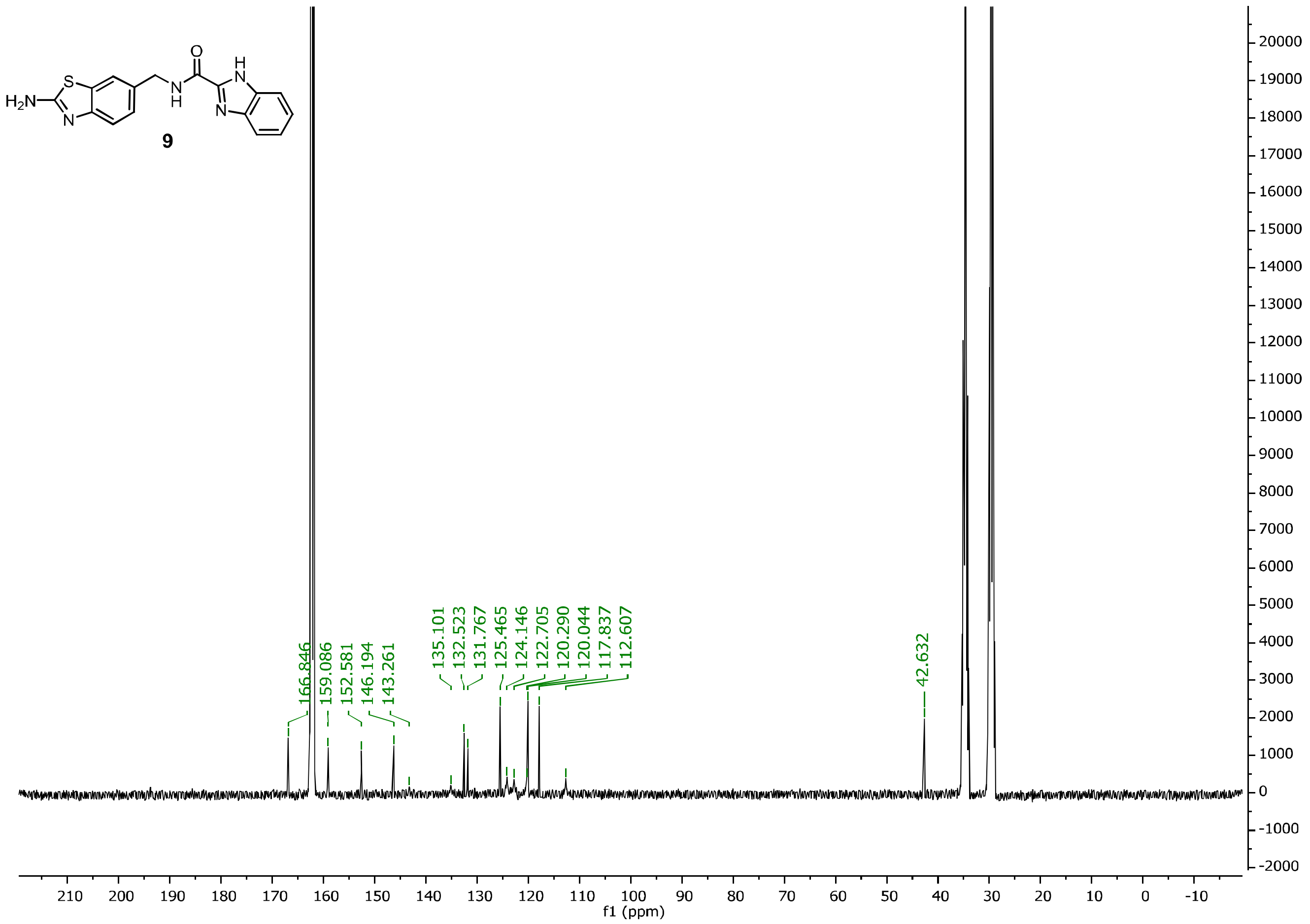

H2N
S
N
O
N
H
H
N
N
9
166.846
159.086
152.581
146.194
143.261
135.101
132.523
131.767
125.465
124.146
122.705
120.290
120.044
117.837
112.607
42.632
20000
19000
18000
17000
16000
15000
14000
13000
12000
11000
10000
9000
8000
7000
6000
5000
4000
3000
2000
1000
0
-1000
-2000
210 200 190 180 170 160 150 140 130 120 110 100 90 80 70 60 50 40 30 20 10 0 -10
f1 (ppm)
